# A-DVM: A Self-Adaptive Variable Matrix Decision Variable Selection Scheme for Multimodal Problems

**DOI:** 10.3390/e22091004

**Published:** 2020-09-09

**Authors:** Marco Antonio Florenzano Mollinetti, Bernardo Bentes Gatto, Mário Tasso Ribeiro Serra Neto, Takahito Kuno

**Affiliations:** 1 School of Systems and Information Engineering, University of Tsukuba, Tsukuba 305-8577, Japan; takahito@cs.tsukuba.ac.jp; 2 Center for Artificial Intelligence Research (C-AIR), University of Tsukuba, Tsukuba 305-8577, Japan; bernardo@cvlab.cs.tsukuba.ac.jp; 3 Department of Computer Science, University of Porto, 4050-290 Porto, Portugal; mariotrsn@gmail.com

**Keywords:** artificial bee colony, swarm intelligence, multimodal problems

## Abstract

Artificial Bee Colony (ABC) is a Swarm Intelligence optimization algorithm well known for its versatility. The selection of decision variables to update is purely stochastic, incurring several issues to the local search capability of the ABC. To address these issues, a self-adaptive decision variable selection mechanism is proposed with the goal of balancing the degree of exploration and exploitation throughout the execution of the algorithm. This selection, named Adaptive Decision Variable Matrix (A-DVM), represents both stochastic and deterministic parameter selection in a binary matrix and regulates the extent of how much each selection is employed based on the estimation of the sparsity of the solutions in the search space. The influence of the proposed approach to performance and robustness of the original algorithm is validated by experimenting on 15 highly multimodal benchmark optimization problems. Numerical comparison on those problems is made against the ABC and their variants and prominent population-based algorithms (e.g., Particle Swarm Optimization and Differential Evolution). Results show an improvement in the performance of the algorithms with the A-DVM in the most challenging instances.

## 1. Introduction

Artificial Bee Colony (ABC) is a swarm intelligence (SI) heuristic for optimization problems inspired by the foraging behavior of honeybees. It was initially designed to solve box-constrained continuous problems [1]. The algorithm consists of three main steps–employed bees, onlooker bees and scout bees–that perform local and global search. In the original implementation of the ABC, at the employed and onlooker bees steps, a single component from each solution is chosen to be updated by a position update rule.

Following its conception, improvements to the search capabilities of the original ABC were proposed by many researchers. The great majority of these propositions centered around changes to the initialization of solutions in the solution space, update procedure of the first two phases and selection method in the onlooker phase [2]. Despite differences between each variant, they all share a common trait: the solution update rule chooses one to *n* decision variables with equal probability under a random uniform distribution. This may allow for a better exploration of the search space and prevent solutions to collapse in the same subspace at later iterations. However, issues to the consistency and convergence of the algorithm may arise due to this design choice.

Besides the uniformity of choice of decision variables, it has been observed that most ABC variants handle problems whose objective functions are multimodal poorly. Adaptations of the ABC to solve problems from this family include changes to the main update equation; adoption of a self-adaptive solution set growth/shrink scheme; and adaptations to the re-sampling step of stagnated solutions [2]. Despite efforts, the ABC still lacks a way to understand how the solutions are fitted and how apart they are in the objective function landscape.

Taking into account the deficiencies observed in the ABC mentioned above, we propose the Adaptive Decision Variable Matrix (A-DVM), a self-adaptive decision variable selection procedure that is an extension of a deterministic solution variable scheme developed in Mollinetti et al. [3]. A-DVM builds an augmented binary matrix that automatically balances deterministic and random decision variable selection to maintain a healthy amount of exploration in the early iterations while emphasizing exploitation in later stages. Levels of exploration and exploitation are monitored by an indicator of how many solutions cover the search space. The chosen estimator is the Δ value, a measure proposed by Morrison [4], which provides a reliable assessment of the shape of the distribution of the solutions along the main and peripheral axes of search. To validate the proposed approach, A-DVM is incorporated into the original ABC and several state-of-the-art variants and evaluated in a test set that features 15 multimodal unconstrained problems. Results are compared to the original counterparts of the ABCs, as well as to some well-established optimization algorithms such as the Particle Swarm Optimization (PSO) and Differential Evolution (DE).

The contributions that the A-DVM brings is twofold. First, a selection scheme that attempts to establish a balance between the global and local search along with the iterations so that the search can be conducted more efficiently. Naturally, it can be incorporated into any version of the ABC without interfering with any other mechanism. Second, a mean to assess how solutions of the solution set are spread throughout the search space and how well they fit the objective function value landscape. Such information is very beneficial in guiding solutions out of deceptive local optima when considering multimodal problems.

This work is organized as follows: Section 2 describes the original ABC. Section 3 discusses the main issues behind the fully randomized selection. Section 4 explains the idea behind the A-DVM. Section 5 reports the experiments and discusses the results. Lastly, Section 6 outlines the conclusion of the paper and points some future directions.

## 2. Artificial Bee Colony

Artificial Bee Colony (ABC) is a Swarm Intelligence (SI) heuristic developed by Karaboga [1] based on the mathematical model of the foraging and information sharing behavior of honey bees. The popularity of the ABC is due to its simple design and easiness to adapt to other families of optimization problems [5]. The conception of the algorithm was to solve continuous optimization problems of the following form:

(1) $$\begin{array}{cc} \mathrm{minimize} & \;f\left(x\right)\\ \mathrm{subject}\;\mathrm{to} & \;{\underline{x}}_{j}\leq x_{j}\leq \bar{x_{j}},j=1,\ldots ,d, \end{array}$$

where $f:R^{n}\to R$ is assumed to be multimodal. The canonical ABC described in Algorithm 1 is composed of four main steps, initialization, employed bees, onlooker bees, and scout bees step. In the initialization step, the solution set *X* is initialized based on specific rules. Then, solutions are sampled and updated by local and global search procedures iteratively until a stopping criterion is met.

ABC has three tunable parameters, the solution set size SN; the maximum number of iterations MCN; and the solution stagnation threshold Lit. A brief description of each step is given below. Let $X=\{x^{1},x^{2},\ldots ,x^{SN}\}$ be the solution set where each $x^{i}$ is a point in $R^{d}$.
 **Algorithm 1:** Canonical Artificial Bee Colony 
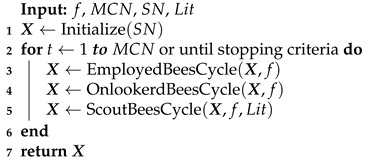



### 2.1. Initialization

If no information of the solution space is provided, $x^{i}$ is sampled from a uniform distribution in the feasible interval $\left[{\underline{x}}_{j},\bar{x_{j}}\right]$ of each decision variable $x_{j}^{i}$, i.e., for i=1,2,…,SN,

(2) $$x^{i}={\underline{x}}_{j}+U(0,1)\left(\bar{x_{j}}-{\underline{x}}_{j}\right),j=1,\ldots d.$$

where U(0,1) denotes a uniform distribution between 0 and 1. A counter $l_{i}=0$ to indicate unsuccessful updates is initialized for each *i*.

### 2.2. Employed Bees Cycle

A randomly chosen component $x_{j}^{i}$ of each solution $x^{i}\in X$ is moved by a random step size towards the *j*th component of some $x^{k}\in X,\;k\neq i$. Therefore $x^{i}$ is updated into:

(3) $$x_{q}^{i}=\left\{\begin{array}{cc} x_{j}^{i}+\phi \;\left(x_{j}^{i}-x_{j}^{k}\right) & \mathrm{if}\;q=j,\\ x_{q}^{i^{\prime }}, & \mathrm{otherwise}, \end{array}\right.$$

where ϕ∈U(−1,1). To verify if the update step was successful, the value of *f* is evaluated and a greedy selection is done for each i=1,2…,SN:

(4) $$x^{i}=\left\{\begin{array}{cc} x^{i}+\left(x_{j}^{i^{\prime }}-x_{j}^{i}\right)e_{j},\;l_{i}=0 & \mathrm{if}\;f\left(x^{i}+\left(x_{j}^{i^{\prime }}-x_{j}^{i}\right)e_{j}\right)\leq f\left(x^{i}\right),\\ x^{i},\;l_{i}=l_{i}+1 & \mathrm{otherwise}, \end{array}\right..$$

where $e_{j}$ is the *j*-th fundamental vector. Needless to say, if (3) fails, then (4) will flag (3) as a failed update.

### 2.3. Onlooker Bees Phase

The solution $x^{i}\in X$ is chosen with probability $p_{i}$ according to a weighted roulette selection scheme and updated using (3). This step can be thought of enhancing local search for solutions with better objective function values. The probability $p_{i}$ is determined for each solution $x^{i}\in X$ as follows,

(5) $$selectfontp_{i}=\frac{F(x^{i})}{\sum_{i=1}^{SN}F\left(x^{i}\right)},$$

where F(·) is the adjusted objective function value:

(6) $$F\left(x^{i}\right)=\left\{\begin{array}{cc} \frac{1}{1+f(x^{i})} & \mathrm{if}\;f(x^{i})\geq 0\\ \left|1+f(x^{i})\right| & \mathrm{otherwise}. \end{array}\right.$$

### 2.4. Scout Bees Phase

Let $\tilde{X}=\left\{x^{k}\in X\;|\;l_{k}\geq Lit\right\}$ denote the set of solutions flagged as stagnated. A new point in $R^{d}$ is resampled using (2) if $\tilde{X}\neq \emptyset$. This prevents the algorithm from premature convergence to bad local optima and increases the number of explorations. The parameter Lit is commonly defined equal to SN·d. If $\tilde{X}\neq \emptyset$, then $x^{w}\in \mathrm{argmax}\left\{f\left(x\right)\;|\;x\in \tilde{X}\right\}$ is always chosen to be resampled.

### 2.5. ABC Variants

Due to the modular nature of the ABC it is easy to make changes to any of the three steps of the algorithm [6]. Many modifications were proposed by several researchers to improve on some difficulties of the algorithm such as inclusion of memory to assist local search; efficient mechanisms to displace solutions stuck in local optima; handle high-dimensional (d>100) problem instances; changes to the update rule; and initialization of solutions using local information. As observed by Aydin et al. [2], the ABC variants often differ in some core components such as the initialization of the first solution set; the update step in the employed and onlooker bees; the computation of selection probabilities in the onlooker bees, the way of displacing solutions in the scout step.

Some well-known and successful variants include the chaotic ABC version of Alatas and Bilal [7] which uses chaotic maps for solution initialization, the ABC of Akay and Karaboga [8] and Gao and Liu [9] which update multiple decision variables in a single update step. Additionally, the ABC with modified selection scheme based on neighborhood distances by Diwold et al. [10], integration of the Differential Evolution algorithm with the ABC by Xiang et al. [11] and Akay et al. [12] are well known. The reader is encouraged to read the survey of Karaboga et al. [6], Sharma and Bhambu [13] for further information.

## 3. Issues Of Randomization

Population-based optimization methods usually employ randomization. By choosing step sizes, decision variables or even target solutions at random during the update steps, population-based optimization methods can “cover more ground” in the search space effortlessly. This is a key element to the success of population-based heuristics, but not without some unintended side effects.

For the sake of clarity, we refer in accordance to [14] to a neighborhood N(·) as the classical definition of a Euclidean ball centered at a point $x^{k}$,

(7) $$selectfontN\left(x^{k}\right)=\left\{x\in R^{d}:{∥x-x^{k}∥}_{2}\leq \epsilon \right\},$$

where ${∥\cdot ∥}_{2}$ is the $\ell_{2}$ norm and ϵ≥0. Assume that a stochastic heuristic, such as the Artificial Bee Colony (ABC), runs infinitely on a problem (f,S)∈P, where *f* is the objective function, *S* is the feasible set, and P is a problem family. Moreover, borrowing some concepts explained in [15], let $X_{f,S}\left(\omega \right)=\left\{f\left(\omega_{k}\right)\;|\;k=1,2,\ldots \right\}$ denote the infinite sequence of iterates generated by the heuristic where $\omega =\{\omega_{k}\;|\;k=1,2,\ldots \}$ is a sequence of random numbers distributed independently from (f,S). Let $X_{f,S}^{\prime }$ and ${\bar{X}}_{f,S}$ denote the set of accumulation points and the closure of the sequence $X_{f,S}\left(\omega \right)$. Lastly, let $X_{f,S}^{*}$ denote the set of global optima. Clearly no $X^{*}$ can be “seen” if ${\bar{X}}_{f,S}\cap X_{f,S}^{*}=\;\emptyset$ or ${\bar{X}}_{f,S}^{\prime }\cap X_{f,S}^{*}=\;\emptyset$.

In the following section, we will see how randomization affects the performance of the ABC.

### An Analysis of the ABC Decision Variable Selection

Often overlooked, a common aspect of the ABC variants is that decision variable $x_{j}^{i}$ is chosen according to the same uniform distribution with for all j=1,2…,d during the Onlooker and Employed bees steps.

Let $Pr(x_{j}^{i})$ be the probability that $x_{j}^{i}$ is chosen in (3). For each situation below, we assume that $\left(f,S\right)\in P,\;{\bar{X}}_{f,S}⋂X_{f,S}^{*}\neq \emptyset$ and $X_{f,S}\left(\omega \right)$ is monotonically decreasing, i.e., $f\left(\omega_{1}\right)\geq f\left(\omega_{2}\right)\geq \ldots$. The original ABC chooses a single $x_{j}^{i}$ each time it calls (3) during the Onlooker and Employed bees step. We need to notice the following issues brought by the process of selecting *j* randomly.

1. **Failed update steps cause solutions to be trapped in basins of attraction:** Choosing the same wrong decision variable many times fails to move solutions out of basins of attraction, contributing to wasteful iterations, premature convergence and needless flagging of solutions at the scout bees step. Let $x^{w^{*}}\in X_{f,S}^{\prime },x^{k^{*}}\in X_{f,S}^{\prime },x^{s^{*}}\in X_{f,S}^{\prime }$. Suppose that $x^{w}\in N\left(x^{w^{*}}\right)$ and $x^{k}\in N\left(x^{k^{*}}\right)$. Also suppose $f\left(x^{w}\right)=f\left(x^{w^{*}}\right)$, $f\left(x^{w}\right)\approx f\left(x^{s^{*}}\right)$, $f\left(x^{w}\right)>f\left(x^{k^{*}}\right)$, and $N(x^{k^{*}})$ and $N(x^{s^{*}})$ are adjacent to $N(x^{w^{*}})$.
  Lastly, let $x_{j}^{w}$ be a component of $x^{w}$ such that a successful update moves $x^{w}$ to $N(x^{k^{*}})$ while an update to $x_{q}^{w}$ for any q≠j moves $x^{w}$ to $N(x^{s^{*}})$. $x^{w}$ is moved in (3) one axis at a time, if $x_{j}^{w}$ is chosen, then (4) accepts $x^{w^{\prime }}$ and $l_{w}=0$. Otherwise, $l_{w}$ is incremented by 1 every time $x^{w^{\prime }}$ is rejected by (4). If each component is chosen in (3) with equal probability, then the probability of $x_{j}^{w}$ to be chosen is $Pr(x_{j}^{w})=1/d$. Therefore, $x^{w}$ has a probability of 1−1/d to move to a basin of attraction $N(x^{s^{*}})$ similar to $N(x^{w^{*}})$, and probability 1/d to move to a more promising region $N(x^{k^{*}})$.
2. **Decision variables may never be chosen:** If the problem is of high-dimensional (d>100) or the evaluation function f(x) is so expensive that only a limited number of objective function calls are allowed, there will be at least a component $x_{j}^{i}$ that may never be chosen in (3). Let $Pr(∼x_{j}^{i})=1-1/d$ be the probability of $x_{j}^{i}$ *not* to be chosen at (3). Then the probability of $x_{j}^{i}$ *not* to be chosen be at the end of MCN iterations is $Pr_{MCN}\left(∼x_{j}^{i}\right)=\prod_{n=1}^{2MCN}\left(1-1/d\right)$. It is clear that $Pr_{MCN}\left(∼x_{j}^{i}\right)$ converges to 0 as the number of iterations goes to infinity. If d>100 and ABC runs t≈d iterations, then $Pr_{MCN}\left(∼x_{j}^{i}\right)\ll 1$, so $x_{j}^{i}$ is not chosen in (3).

At first glance, there would be two ways to resolve these issues. Either assign a non-equal probability to choose the decision variable or choose more than one $x_{j}^{i}$ at (3) to be updated simultaneously. We disprove the effectiveness of these “quick fixes” through following arguments.

- Changing the choice probabilities of decision variables to be unequal would not solve issue #2 in high-dimensional problems because $Pr_{MCN}\left(∼x_{j}^{i}\right)$ still converges to 0. A sufficient measure, in this case, would keep previously chosen components in memory. This only increases the complexity of the Onlooker and Employed bees phase from O(n) to O(nlogn) if non-visited components are kept in a separate list for each solution in *X* in an efficient way
- Changing (3) to choose multiple components from $x^{i}$ would not improve issue #1. Let J⊂{1,…,d} and suppose that $x_{j}^{i}$ is chosen for each j∈J to update. Update rule (3) is an affine transformation in the *j*-th axis along the line segment between $x_{j}^{i}$ and $x_{j}^{k},\;i\neq k$. If |J|>1, then |J| simultaneous affine transformations in the |J|-dimensional subspace between $x^{i}$ and $x^{k}$ would be performed. In terms of complexity, there would be no burden *j* decision variables are updated at once by means of a matrix product operation. However, in terms of performance, there would be no improvement because of two reasons. First, moving along many axes at once does not reduce the possibility of $x^{i}$ to remain in $N(x^{*})$ if $x^{k}\in N\left(x^{*}\right)$. Secondly, setting |J|>1 in (3) has been shown to be not as good as |J|=1 in later iterations due to the coarseness of the search when most of the solutions have converged to a single accumulation point [2].

In the following section, we present a method that provides a solution to the issues stated above, the Adaptive Decision Variable Matrix (A-DVM). A-DVM is a decision selection variable scheme proposed to the ABC.

## 4. A Novel Decision Variable Selection Mechanism

We propose a method for selecting decision variables efficiently without any additional memory nor simultaneous update of multiple components. The Adaptive Decision Variable Matrix (A-DVM) is an extension of the decision variable selection procedure of Mollinetti et al. [3]. It exploits the same modular nature as the Artificial Bee Colony (ABC), and thereby it can be integrated to the employed and/or onlooker bees phase without interfering with any additional steps of the original or any variant. To emphasize the difference between the A-DVM and Mollinetti et al. [3] deterministic selection, we briefly explain their proposition as follows.

### 4.1. Fully Deterministic Decision Variable Selection

The selection scheme proposed by Mollinetti et al. [3] is inspired by Cantor’s Diagonalization argument used to prove the non-existence of bijection from the set of natural numbers to the set of real numbers [16,17]. Cantor’s argument state that any binary square matrix *T* does not have the same column as the vector consisting of the complements of the diagonal elements of *T*. The authors extended this notion to generate new solutions $x^{i}$ in the solution set *X*. For any given problem, the deterministic decision variable selection arranges the solution set *X* into a $R^{d\times SN}$ matrix:

$$A=[x_{1}\;x_{2}\;\cdots \;x_{SN}].$$

If *A* is a square matrix, the entries on the main diagonal are stored in an *m*-vector $c={\left(a_{11},a_{22},\ldots ,a_{mn}\right)}^{⊤}$ and undergo the update step. In general, the higher the number of solutions, the better the exploration of the search, and so SN>d holds. If *A* is wide, then vector *c* consists of entries on the main diagonal and the superdiagonals of *A* offset *d* units to the right. For instance, if *A* is a 2×6 matrix, then *c* will be:

$$A=\left[\begin{array}{cccccc} a_{11} & a_{12} & a_{13} & a_{14} & a_{15} & a_{16}\\ a_{21} & a_{22} & a_{23} & a_{24} & a_{25} & a_{26} \end{array}\right]\;c={\left(a_{11},a_{22},a_{13},a_{24},a_{15},a_{26}\right)}^{⊤}.$$

The vector *c* allows (3) to be performed simultaneously for all columns of *A* by means of a simple vector multiplication:

(8) $$selectfontc^{\prime }=\psi \left(c+\Phi ⊙\left(c-z\right)\right),$$

where ⊙ is the Hadamard product, Φ is a SN column vector of values sampled from U(−1,1), $z={\left(z_{1},\ldots ,z_{SN}\right)}^{⊤}\neq c$, and ψ(·) is a function similar to (4) defined as:

(9) $$\psi \left(c_{i}^{\prime }\right)=\left\{\begin{array}{cc} c_{i}^{\prime },\;l_{i}=0 & \mathrm{if}\;f\left(x^{i}+\left(c_{i}^{\prime }-x_{j}^{i}\right)e_{j}\right)\leq f\left(x^{i}\right)\\ c_{i},\;l_{i}=l_{i}+1 & \mathrm{otherwise}. \end{array}\right.$$

Suppose in the matrix *A* of the above example that if $f\left(x^{1}+\left(c_{1}^{\prime }-x_{1}^{1}\right)e_{1}\right)\leq f\left(x^{1}\right)$ and $f\left(x^{2}+\left(c_{2}^{\prime }-x_{2}^{2}\right)e_{2}\right)\leq f\left(x^{2}\right)$, then $c^{\prime }={\left(c_{1}^{\prime },c_{2}^{\prime },a_{13},a_{24},a_{15},a_{26}\right)}^{⊤}$. Thus, entries $a_{11}$ and $a_{22}$ are replaced with $c_{1}^{\prime },c_{2}^{\prime }$ in *A*, and the corresponding values of *f* are updated.

Lastly, a safeguard step is performed so that every decision variable of each candidate solution can be updated at least once before the algorithm termination. The last column $x^{SN}$ of *A* is moved to the first position and the remaining columns are shifted one position to the right. Referring to the example, the matrix *A* is now:

$$A=\left[\begin{array}{cccccc} c_{1}^{\prime } & a_{12} & a_{13} & a_{14} & a_{15} & a_{16}\\ a_{21} & c_{2}^{\prime } & a_{23} & a_{24} & a_{25} & a_{26} \end{array}\right]\;⟶\left[\begin{array}{cccccc} a_{16} & c_{1}^{\prime } & a_{12} & a_{13} & a_{14} & a_{15}\\ a_{26} & a_{21} & c_{2}^{\prime } & a_{23} & a_{24} & a_{25} \end{array}\right]$$

This step ensures that every decision variable is updated by (8) every *d* iterations.

The results in Mollinetti et al. [3] indicate that eliminating the randomness in the choice of the decision variable in (3) boosted the performance of the original ABC in multimodal problems of up to 30 decision variables. However, it is observed that the diversity of solutions was compromised because local search was more emphasized over global search. From this result, we suppose that the bias towards local search brought by the fully deterministic parameter selection has yet not solved **issue #1**. In fact, if anything, the fully deterministic selection made it worse. Therefore, reintroducing a small degree of randomness while guaranteeing that every solution is chosen at some iteration is a step in the right direction to refocus global search.

### 4.2. A Self-Adaptive Decision Variable Selection Procedure (A-DVM)

Let us change the focus to a partially deterministic selection, and reintroduce an adaptive degree of randomness to the selection process based on the “spread” of solutions throughout the search space. The variables $x_{j}^{i}$ are chosen via a binary decision matrix. The goal of the A-DVM is not only to provide an acceptable solution to the issues discussed in Section 3, but to improve the overall performance of the state-of-the-art of ABC for the multimodal and high-dimensional problem of the form of (1).

The main piece of the A-DVM is the d×SN binary matrix $P_{am}$ that represents which $x_{j}^{i}$ has been chosen to be updated by (3) or (8). The matrix $P_{am}$ is a composition of two matrices, $P_{r}$, a binary matrix with a single 1 in each column, whose row is determined randomly according to a uniform distribution; and $P_{d}$, a matrix with 0 or 2 in each entry generated by the fully deterministic scheme of Mollinetti et al. [3]. For example, $P_{r}$ and $P_{d}$ are matrices of the form:

$$P_{r}=\left(\begin{array}{cccccccc} 0 & 0 & 0 & \ldots & 1 & 0 & \ldots & 0\\ 0 & 0 & 1 & \ldots & 0 & 0 & \ldots & 0\\ 1 & 0 & 0 & \ldots & 0 & 1 & \ldots & 0\\ \vdots & \vdots & \vdots & \ddots & \vdots & \vdots & \ddots & 1\\ 0 & 1 & 0 & \ldots & 0 & 0 & \ldots & 0 \end{array}\right),\;\;\;\;\;\;\;\;P_{d}=\left(\begin{array}{cccccccc} 2 & 0 & 0 & \ldots & 0 & 2 & \ldots & 0\\ 0 & 2 & 0 & \ldots & 0 & 0 & \ldots & 0\\ 0 & 0 & 2 & \ldots & 0 & 0 & \ldots & 0\\ \vdots & \vdots & \vdots & \ddots & \vdots & \vdots & \ddots & \vdots \\ 0 & 0 & 0 & \ldots & 2 & 0 & \ldots & 2 \end{array}\right).$$

The matrix $P_{am}$ is the result of a composition of $P_{r}$ into $P_{d}$. That means some solutions $x_{i}\in X$ have their *j*-th component randomly selected when updated by (3) or (8) while the rest have their *j*-th component chosen by the fully deterministic scheme. We write $P_{am}=\beta P_{r}⊕\alpha P_{d}$ when β% of the columns of $P_{am}$ are from $P_{r}$ and the remaining α(=1−β)% are from $P_{d}$. An example of $P_{am}$ based on the above example is as follows:

$$\begin{array}{c} P_{am}=\beta P_{r}⊕\alpha P_{d}=\left(\begin{array}{cccccccc} 0 & 0 & 0 & \ldots & 0 & 0 & \ldots & 0\\ 0 & 2 & 0 & \ldots & 0 & 0 & \ldots & 0\\ 1 & 0 & 2 & \ldots & 0 & 1 & \ldots & 0\\ \vdots & \vdots & \vdots & \ddots & 0 & \vdots & \ddots & 0\\ 0 & 0 & 0 & \ldots & 2 & 0 & \ldots & 2 \end{array}\right). \end{array}$$

The degree of how much $P_{d}$ is favored over $P_{r}$ is represented by the coefficient α that is iteratively adjusted as follows, to maintain a healthy diversity of solutions while balancing between local search and local search:

(10) $$selectfont\alpha =\left(1-\Delta \right)K_{1}+\Delta K_{2},$$

where Δ∈[0,1] is the measure of the dispersion of the population at the current iteration and $K_{1}$ and $K_{2}$ are scaling parameters set to 0.3 and 0.7 in accordance to McGinley et al. [18]. Values of α close to 1 signify high population diversity and activate exploitation by the deterministic selection. On the other hand, values close to 0 boost exploration using random selection. Because solutions in population-based algorithms tend to concentrate around accumulation points $x^{\prime }\in X_{f,S}^{\prime }$ after a considerable amount of iterations [14], α is increased by a growth function ρ defined as follows, to intensify local search around $x^{\prime }$ after $t^{\prime }$ iterations:

(11) $$\rho \left(\cdot \right)=\left\{\begin{array}{cc} \alpha =\alpha e^{\gamma t} & \mathrm{if}\;t>t^{\prime },\\ \alpha & \mathrm{otherwise}, \end{array}\right.$$

where γ is set to 0.01. The value of $t^{\prime }$ is given by:

(12) $$selectfontt^{\prime }=\min\left(\frac{n\cdot d}{\lambda_{t}\cdot t_{max}},\;\lambda_{t}t_{max}\right),$$

where an acceptable value for $\lambda_{t}$ was empirically verified to be 0.1.

To ensure that every decision variable is chosen in at least every *d* iterations, we introduce a history $H\in {\left\{0,1\right\}}^{d}$ that stores which columns of $P_{d}$ were put into $P_{am}$, and give a chance to the remaining columns of $P_{d}$ to be contained in $P_{am}$ at the next iteration. We enforce a bound on the number of iterations that solutions are chosen by the fully deterministic selection to be no more than $\frac{3}{5}K_{1}$ and no less than $\frac{1}{2}K_{2}$ (refer to (10)). When the entries in ***H*** are all ones, ***H*** is reinitialized and the whole process runs again. The overall steps of the A-DVM are outlined in Algorithm 2.
 **Algorithm 2:** Steps of the A-DVM 
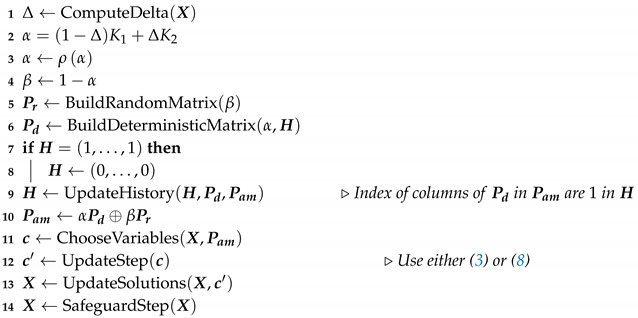


### 4.3. The Δ Dispersion Estimate

Estimating the dispersion of the solutions in the search space is specifically effective for population-based algorithms to deal with multimodal or high-dimensional problems. Measuring how far apart solutions in ***X*** are from each other is very helpful to guide them towards accumulation points or free them from local optima. Significant contributions related to this subject can be found in Ursem [19] and Back et al. [20] which introduced the Sparse Population Diversity (SPD) metric, a method for estimating the variation of the solution set by measuring the distance from each solution in relation to the centroid. McGinley et al. [18] proposed the Healthy Population Diversity (HPD), an extension of the SPD that introduces the concept of individual contribution to the computation of the centroid.

Metrics like SPD and HPD may accurately and inexpensively identify differences between the solutions in ***X*** by measuring the distances to each $x^{i}$. However, this kind of measurement does not take into account how the solutions are distributed in the search space, which is problematic since the same measurement values from SPD and HPD may indicate different search-space coverage of solution of ***X***. Because of that, we employ the Δ dispersion measure introduced in Morrisson [4], initially proposed for Evolutionary Algorithms with binary solution encoding, and adapt it to continuous problems in the form of (1). Computation of Δ is as follows:

(13) $$selectfont\Delta =\Delta_{1}+\Delta_{2}=\frac{1.75-S}{1.75},$$

where $\Delta_{1}=0.75-S_{1}$, $\Delta_{2}=1-S_{2}$ and $S=S_{1}+S_{2}$. The values of $S_{1}$ and $S_{2}$ are obtained by measuring the moment of inertia of the solution centroid in relation to each solution. We denote as $P=\left|X\right|$ as the number of solutions $x^{i}\in X$. The centroid $cr_{j}$ of the *j*th components and the moment of inertia $I_{j}$ of centroid $cr_{j}$ are

(14) $$selectfontcr_{j}=\frac{\sum_{i=1}^{P}x_{j}^{i}}{P},\;I_{j}=\sum_{i=1}^{P}{\left(x_{j}^{i}-cr_{j}\right)}^{2},\;j=1,\ldots ,d.$$

The first measure $S_{1}$ involves a quantitative assessment of the solutions around the distribution centroid. Assuming the distribution around the centroid to be uniform, $S_{1}$ is

(15) $$selectfontS_{1}=\max_{j}\frac{\left[\left|I_{U_{o}}-I_{j}+Pcr_{j}^{2}\right|\right]}{P},$$

where $I_{U_{o}}$ represents the inertia of a uniform distribution:

(16) $$selectfontI_{U_{o}}=\sum_{i=1}^{P}{\left(\frac{i}{P+1}\right)}^{2}.$$

Measure $\Delta_{2}$ indicates how much the calculation of $\Delta_{1}$ is misleading when the distribution is not uniform in the search-space, since $\Delta_{1}$ only verifies non-uniformity along the principal diagonal of the search space. The second measure $S_{2}$ is

(17) $$S_{2}=\max\left[\frac{\left|\sum_{P}\chi_{c+}-\left\lceil \left(\frac{\prod_{j}\left(1-cr_{j}\right)}{\prod_{j}\phi_{j}}\right)P\right\rceil \right|}{P},\frac{\left|\sum_{P}\chi_{c-}-\left\lceil \left(\frac{\prod_{j}\left(cr_{j}\right)}{\prod_{j}\phi_{j}}\right)P\right\rceil \right|}{P}\right].$$

where $c-=\left\{x_{j}^{i}\in X|x_{j}^{i}\leq cr_{j}\right\},c+=\left\{x_{j}^{i}\in X|x_{j}^{i}\geq cr_{j}\right\}$, χ is the characteristic function that returns either 0 or 1 whether a solution belongs to c− or c+, respectively, and $\phi_{j}$ is the range between $\left[{\underline{x}}_{j},{\bar{x}}_{j}\right]$, so that $\prod_{j=1}^{d}\phi_{j}=1$ for an *N*-dimensional unit volume.

### 4.4. Remarks On Complexity

As for the complexity, SN function evaluations are done in each employed and onlooker bees phase, so the addition of A-DVM preserves the same 2n+1 function evaluations per iteration as the classical ABC. The effort to compute the sum of moments of inertia and Δ dispersion is proportional to the solution set size SN [4]. Updates of solutions (3) during the employed or onlooker bees is done one by one in a loop require O(n) time for the size SN of solution set ***X***. On the other hand, offline update (8) can be done in a linear time due to the vector multiplication. We recommend (8) for parallel versions of the ABC, when MCN is large or the evaluation of f(x) is expensive.

Regarding lookup table ***H***, it is verified in O(n) time which columns of $P_{d}$ were not chosen to be a part of $P_{am}$. Lastly, about binary matrix $P_{d}$, because the deterministic parameter selection extracts the diagonal of solution set ***X***, it is recommended to set SN≥d to ensure that every decision variable of each solution is chosen in at most *d* iterations.

## 5. Experiment and Results

A numerical experiment was carried out to answer the following research questions: “Does incorporating the Adaptive Decision Variable Matrix (A-DVM) improve the Artificial Bee Colony (ABC) and its variants overall performance on multimodal problems?”. To provide an answer to that question, we chose 15 instances of (1), each of which is designed to validate the capability of metaheuristics to handle multimodal and non-smooth objective functions. The instances are ranked in the top 30 hardest continuous optimization functions in the Global Optimization Benchmarks suite [21]. The number of variables ranges from 2 to 30 to test the robustness of the solvers when dealing with many as well as few variables. Each algorithm is executed 30 times with the same seed interchangeably in a random fashion to avoid bias in the machine load. The number of variables, the box constraint range $\left[l_{j},u_{j}\right]$ and the global optimum of each instance are listed in Table 1.

Testing involves the incorporation of the A-DVM to the onlooker and employed bees phase of the following versions of the ABC: the original ABC from Karaboga [22] (ABC+A-DVM), two versions of the global best guided ABC (gbestABC) from Gao et al. [23] (GBESTABC+A-DVM, GBESTABC2+A-DVM) and two versions of the ABC-X from [2] for multimodal problems (ABC-XM1+A-DVM, ABC-XM2+A-DVM). The original counterparts were also used for the baseline (ABC, GBESTABC, GBESTABC2, ABC-XM1, ABC-XM5) together with the modified ABC for multidimensional functions (MABC) from Akay and Karaboga [8] and its version with the A-DVM (MABC+A-DVM). Comparison is not limited only to ABCs and variants, but popular population-based algorithms, such as the Particle Swarm Optimization from Kennedy and Eberhart [24], Evolutionary Particle Swarm Optimization by Miranda and Fonseca [25] and Differential Evolution (DE) [26], were also included in the experiment.

The stopping criteria for each algorithm was set to $10^{5}$ function evaluations (FE’s) or if the difference between the best value found so far and the global optimum $f(x^{*})$ is less than $10^{-8}$. The population size was common to all algorithms and fixed at 30. For PSO, the inertia factor ($w_{1}$) was set to 0.6 and both cognitive and social parameters ($w_{2}$, $w_{3}$) to 1.8. For Differential Evolution (DE) [26] with *best1bin* strategy, *F* value was 0.5 and *CR* 0.9. For each version of the ABC: Lit=SN·d. For MABC, MR, SF and *m* were 0.5, 0.7, and 2.5% of maximum FE’s, respectively. ABC-X parameters were Lit=1.06·d, maximum population of 66 and minimum of 15 for ABC-Xm1 and Lit=0.83·d, while for ABC-Xm5, maximum population of 78 and minimum of 17. Lastly, parameters γ and $\lambda_{t}$ of the A-DVM were set to 0.1.

The experiment was conducted in a machine with the following hardware configuration: Intel core i7-6700 “Skylake” 3.4 GHz CPU; 16 GB RAM DDR4 3200 clocked at 3000 MHz. The running operating system (OS) is UbuntuOS 18.04. All algorithms were written in the python 3 programming language. Floating point operations were handled by the numpy package, version 1.19.1.

**Figure 1:**
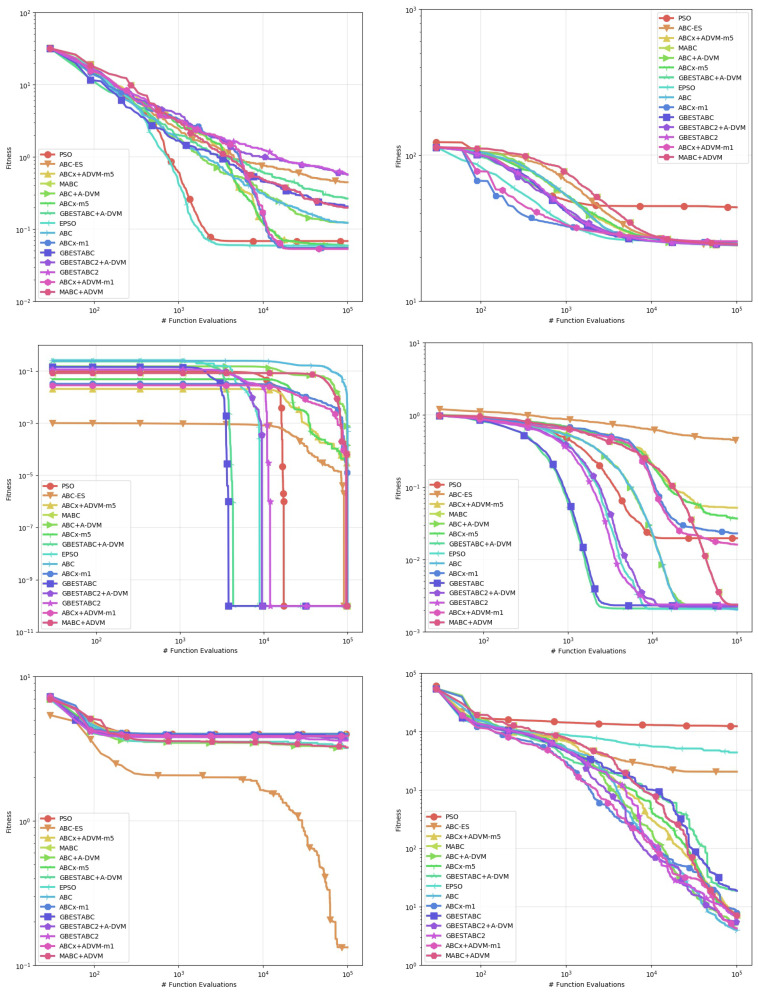
Behavior of the Mean of all executions for Bukin06 to DeVilliersGlasser02 instances.

**Figure 2:**
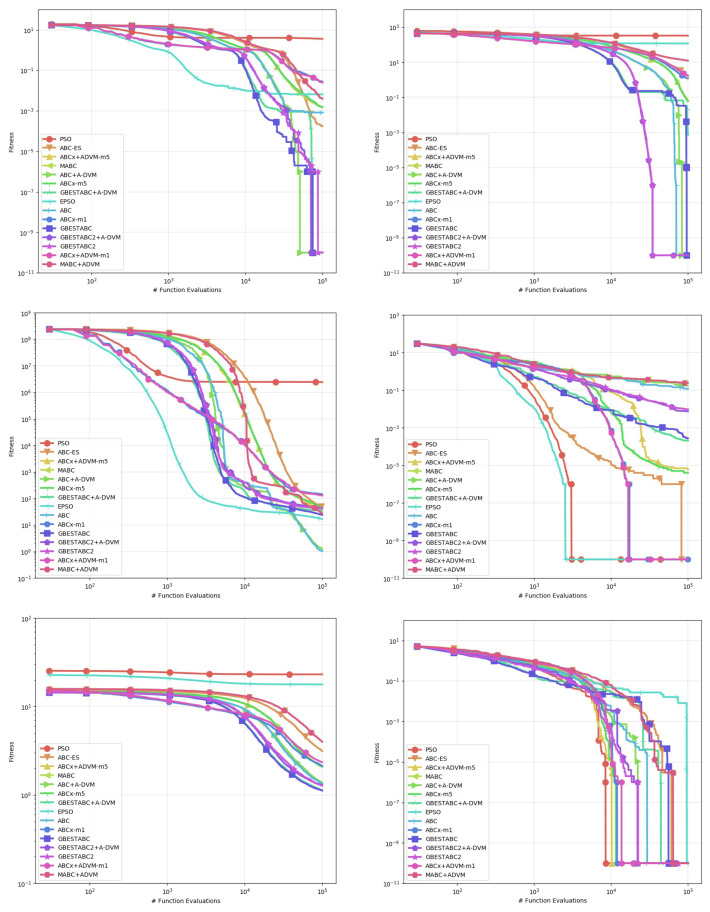
Behavior of the Mean of all executions for Griewank to Trefethen instances.

**Figure 3:**
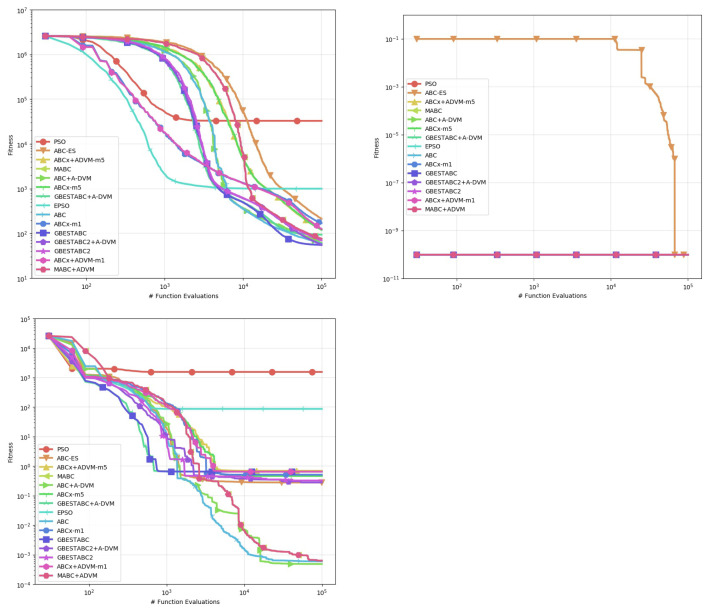
Behavior of the Mean of all executions for Whitley to Zimmerman instances.

Table 2, Table 3, Table 4, Table 5, Table 6 and Table 7 show the computational results obtained from this experiment. The statistics used for comparison are the mean, standard deviation, median, and best-worst results obtained from 30 runs with distinct random seeds shown in Table 6 and Table 7. Statistical significance between pairs is verified by the Mann-Whitney U-test for non-parametric data, with confidence interval α set to 0.95 as shown in Table 2, Table 3, Table 4 and Table 5. Entries where p>0.05 denotes no statistical difference between the algorithms. For better legibility, the precision of decimals is set to 5 digits and values lower than $10^{-6}$ are rounded to 0. Plots of the behavior of each algorithm are shown in Figure 1, Figure 2 and Figure 3. Each line represents the mean of the best solution of all executions for each function evaluation call. All plots were log-scaled for better legibility. If the performance of any algorithm for a particular instance is statistically significant, it means that its *p*-value in the U-test is less than 0.05 in the pairwise comparison against all other algorithms. The bold numbers in the tables indicate the least value for that particular statistic and instance.

First, we discuss the Rosenbrock, Whitley and Zimmerman function instances where the A-DVM resulted in overall worse performance than all their original counterparts. The A-DVM was indeed able to guide the functions towards a valley, but a thorough local search mechanism was lacking due to the parabolic surface of the Rosenbrock function [27]. The same behavior is observed in the Whitley and Zimmerman functions, which share the same property as Rosenbrock instance. The poor results in these functions imply a failure of the A-DVM to properly address issues #1 and #2 discussed in Section 3. Additionally, we can relate this case to the no-free-lunch theorem of Wolpert [28], saying that no algorithm can be strictly better than the others in every problem instance. The inferior results of the A-DVM are also seen for the Rastrigin function in the ABC-X variants. The Cause of such behavior could be due to intensification of the local search mechanism that forced solutions to stay far from the local attractors of the surface of the functions.

Strong evidence of the robustness of the A-DVM against strongly multimodal surfaces was found in the Damavandi, DeVilliersGlasser02 and CrossLegTable instances, ranked as the three hardest functions in the benchmark suite [21]. Both functions feature large basins of attraction for bad local optima, the number of which is directly proportional to the problem dimensionality. There are two possible causes explaining why the A-DVM versions were not superior to all other versions in these particular instances. First, a small number of dimensions means that a square matrix can be built, providing a thorough exploration of the search space. Second, exploration in the early stages allowed solutions to escape from the basins of attraction.

Evidence that the A-DVM improved the search process in comparison to their counterparts without the search can be seen in the Bukin06, SineEnvelope, CrownedCross and Schwefel06. Although the ABCs with A-DVM were not the best solvers, their robustness was statistically significant in comparison to the versions without A-DVM. Lastly, in the Cola, Griewank, XinSheYang03 and Trefethen, no statistical significance that corroborated that the incorporation of the A-DVM improved or worsened the performance of the original algorithm was found.

## 6. Conclusions

In this paper, a decision variable selection scheme named Adaptive Decision Variable Matrix (A-DVM) was proposed to be incorporated in the Artificial Bee Colony (ABC) algorithm. A-DVM can be incorporated in both employed and or onlooker bees phases and can be used with any variant of the ABC. A-DVM attempts to balance exploration and exploitation throughout the execution of the algorithm by constructing an augmented binary matrix that represents the choice of components of solutions in the solution set. The binary matrix is composed of a deterministic selection binary matrix that chooses matrix diagonals according to the proposal in [3] and another binary matrix whose components were selected by a random uniform distribution. The number of columns to be used from the deterministic matrix is determined by a self-adaptive parameter that is based on the Δ value, a measure of the sparsity of the actual solution set in the search space. Introducing a lookup table of chosen solutions of $P_{d}$ guarantees that every solution is a part of $P_{d}$ in the update step at least once before termination.

Effects of the A-DVM to the performance of the ABC is verified by a numerical experiment including several versions of the ABC with the A-DVM included and their original counterparts. Representative heuristics such as the Particle Swarm Optimization (PSO), Evolutionary Particle Swarm Optimization (EPSO) and Differential Evolution (DE) are included in the experiment to provide a baseline for the results. For the sake of brevity and to narrow the scope of this work, other prominent Swarm Intelligence (SI) algorithms suited to the multimodal family of problem, such as the monarch butterfly optimization (MBO) [29]; earthworm optimization algorithm (EWA) [30]; elephant herding optimization (EHO) [31]; and moth search (MS) algorithm [32], were not part of the experiment.

The results indicate that the A-DVM enhances the ability of the ABC to adapt to highly multimodal functions. However, the elimination of the full global search of the stochastic selection resulted in solutions not converging towards accumulation points that are located in basins, as seen in some instances where the A-DVM performed poorly. Integration with ABC variants with smart restart procedures in the scout bees phase may be a possible direction to improve this issue.

Future works include in-depth sensitivity analysis and integration of the selection mechanism to the state-of-the-art ABC used for optimization competitions and testing on large scale problems, mechanical design and power systems to further investigate the performance of the selection. Moreover, a thorough comparative study of multimodal problems using only SI algorithms including the aforementioned examples is due. Another research direction includes applying the proposed method for weight tuning of shallow networks [33,34]. Such networks may benefit from the proposed optimization mechanism since it tackles small sample size problems featuring rough objective function landscapes.

## Figures and Tables

**Table 1:** Definition of Benchmark functions.

Name	Dim	Range	Opt.	Name	Dim	Range	Opt.
Bukin06	10	[−10, 0]	0.0	Rosenbrock	30	[−30, 30]	0.0
Cola	17	[−4, 4]	11.7464	Schwefel06	30	[−500, 500]	0.0
CrossLegTable	2	[−10, 10]	−1.0	SineEnvelope	20	[−500, 500]	0.0
CrownedCross	2	[−10, 10]	0.0001	Trefethen	2	[−10, 10]	−3.0
Damavandi	2	[0, 14]	0.0	Whitley	2	[−10.24, 10.24]	0.0
DeVilliersGlasser02	5	[1, 60]	0.0	XinSheYang03	20	[−500, 500]	0.0
Griewank	30	[−100, 100]	0.0	Zimmerman	2	[0, 100]	0.0
Rastrigin	30	[−5.12, 5.12]	0.0	–	–	–	–

**Table 2:** Pairwise U-test analysis from Bukin06 to Crowned Cross functions.

Bukin06	ABC-ES	ABCx+ADVM-m5	MABC	ABC+A-DVM	ABCx-m5	GBESTABC+A-DVM	EPSO	ABC	ABCx-m1	GBESTABC	GBESTABC2+A-DVM	GBESTABC2	ABCx+ADVM-m1	MABC+ADVM
PSO	1.4323 × 10${}^{-11}$	0.209922	1.48463 × 10${}^{-8}$	0.000219796	0.128748	1.82244 × 10${}^{-10}$	0.168608	0.000463291	0.173632	5.37741 × 10${}^{-10}$	1.4323 × 10${}^{-11}$	1.4323 × 10${}^{-11}$	0.138314	1.48463 × 10${}^{-8}$
ABC-ES	-	1.50993 × 10${}^{-11}$	5.13867 × 10${}^{-7}$	3.87146 × 10${}^{-10}$	1.50993 × 10${}^{-11}$	6.23853 × 10${}^{-5}$	1.47713 × 10${}^{-11}$	4.0485 × 10${}^{-10}$	1.50993 × 10${}^{-11}$	2.22121 × 10${}^{-7}$	0.0424998	0.018891	1.50993 × 10${}^{-11}$	5.13867 × 10${}^{-7}$
ABCx+ADVM-m5	-	-	2.76643 × 10${}^{-8}$	0.000340655	0.449998	1.30493 × 10${}^{-10}$	0.444124	4.89476 × 10${}^{-5}$	0.214482	9.27889 × 10${}^{-10}$	1.50993 × 10${}^{-11}$	1.50993 × 10${}^{-11}$	0.181611	2.76643 × 10${}^{-8}$
MABC	-	-	-	0.00251642	3.25914 × 10${}^{-9}$	0.0328356	4.17254 × 10${}^{-9}$	0.0025418	4.63014 × 10${}^{-9}$	0.255296	2.5461 × 10${}^{-8}$	1.00761 × 10${}^{-8}$	2.28629 × 10${}^{-9}$	0.18406
ABC+A-DVM	-	-	-	-	5.42735 × 10${}^{-5}$	2.85674 × 10${}^{-6}$	3.23538 × 10${}^{-5}$	0.444024	0.000117432	4.43709 × 10${}^{-5}$	2.66077 × 10${}^{-10}$	8.46204 × 10${}^{-11}$	0.000124434	0.00251642
ABCx-m5	-	-	-	-	-	9.7839 × 10${}^{-11}$	0.46758	2.31892 × 10${}^{-5}$	0.294726	3.05885 × 10${}^{-10}$	1.50993 × 10${}^{-11}$	1.50993 × 10${}^{-11}$	0.255299	3.25914 × 10${}^{-9}$
GBESTABC+A-DVM	-	-	-	-	-	-	1.05577 × 10${}^{-10}$	3.14047 × 10${}^{-6}$	1.18573 × 10${}^{-10}$	0.135349	3.1414 × 10${}^{-6}$	4.42055 × 10${}^{-7}$	8.88454 × 10${}^{-11}$	0.0328356
EPSO	-	-	-	-	-	-	-	3.52994 × 10${}^{-5}$	0.479356	3.2109 × 10${}^{-10}$	1.47713 × 10${}^{-11}$	1.47713 × 10${}^{-11}$	0.392179	4.17254 × 10${}^{-9}$
ABC	-	-	-	-	-	-	-	-	2.04147 × 10${}^{-5}$	7.01841 × 10${}^{-5}$	1.73612 × 10${}^{-10}$	9.77827 × 10${}^{-11}$	1.38593 × 10${}^{-5}$	0.0025418
ABCx-m1	-	-	-	-	-	-	-	-	-	4.44312 × 10${}^{-10}$	1.50993 × 10${}^{-11}$	1.50993 × 10${}^{-11}$	0.378099	4.63014 × 10${}^{-9}$
GBESTABC	-	-	-	-	-	-	-	-	-	-	2.15438 × 10${}^{-8}$	2.09022 × 10${}^{-9}$	4.87515 × 10${}^{-10}$	0.255296
GBESTABC2+A-DVM	-	-	-	-	-	-	-	-	-	-	-	0.353086	1.50993 × 10${}^{-11}$	2.5461 × 10${}^{-8}$
GBESTABC2	-	-	-	-	-	-	-	-	-	-	-	-	1.50993 × 10${}^{-11}$	1.00761 × 10${}^{-8}$
ABCx+ADVM-m1	-	-	-	-	-	-	-	-	-	-	-	-	-	2.28629 × 10${}^{-9}$
**Cola**	**ABC-ES**	**ABCx+ADVM-m5**	**MABC**	**ABC+A-DVM**	**ABCx-m5**	**GBESTABC+A-DVM**	**EPSO**	**ABC**	**ABCx-m1**	**GBESTABC**	**GBESTABC2+A-DVM**	**GBESTABC2**	**ABCx+ADVM-m1**	**MABC+ADVM**
PSO	1.50993 × 10${}^{-11}$	1.50993 × 10${}^{-11}$	1.50993 × 10${}^{-11}$	1.50993 × 10${}^{-11}$	1.50993 × 10${}^{-11}$	1.50993 × 10${}^{-11}$	1.50898 × 10${}^{-11}$	1.50993 × 10${}^{-11}$	1.50993 × 10${}^{-11}$	1.50993 × 10${}^{-11}$	1.50993 × 10${}^{-11}$	1.50993 × 10${}^{-11}$	1.50993 × 10${}^{-11}$	1.50993 × 10${}^{-11}$
ABC-ES	-	0.264891	0.00278497	0.342161	0.19355	0.0481314	0.00348583	0.205955	0.000557128	0.0175683	0.185539	0.0511631	3.79957 × 10${}^{-7}$	0.00278497
ABCx+ADVM-m5	-	-	0.00473413	0.467596	0.380914	0.066727	0.00515681	0.310202	0.00102617	0.0362228	0.432497	0.0481314	5.9684 × 10${}^{-7}$	0.00473413
MABC	-	-	-	0.0103404	0.0217918	0.218821	0.028728	0.0111801	0.038636	0.255299	0.0317663	0.185539	6.23853 × 10${}^{-5}$	0.18406
ABC+A-DVM	-	-	-	-	0.38656	0.148636	0.00473364	0.432497	0.00151697	0.0789878	0.358594	0.103103	8.64516 × 10${}^{-7}$	0.0103404
ABCx-m5	-	-	-	-	-	0.181611	0.00636544	0.432497	0.00201649	0.0789878	0.491154	0.155594	1.24566 × 10${}^{-6}$	0.0217918
GBESTABC+A-DVM	-	-	-	-	-	-	0.0120775	0.103103	0.0103404	0.461721	0.201769	0.420901	2.98528 × 10${}^{-5}$	0.218821
EPSO	-	-	-	-	-	-	-	0.00538076	0.380913	0.0188897	0.00955536	0.00814166	0.409372	0.028728
ABC	-	-	-	-	-	-	-	-	0.00102617	0.0648351	0.497051	0.111286	1.24566 × 10${}^{-6}$	0.0111801
ABCx-m1	-	-	-	-	-	-	-	-	-	0.0242067	0.00291408	0.00720609	0.181611	0.038636
GBESTABC	-	-	-	-	-	-	-	-	-	-	0.0978954	0.438317	7.03343 × 10${}^{-5}$	0.255299
GBESTABC2+A-DVM	-	-	-	-	-	-	-	-	-	-	-	0.170144	5.09384 × 10${}^{-6}$	0.0317663
GBESTABC2	-	-	-	-	-	-	-	-	-	-	-	-	1.13901 × 10${}^{-5}$	0.185539
ABCx+ADVM-m1	-	-	-	-	-	-	-	-	-	-	-	-	-	6.23853 × 10${}^{-5}$
**CrossLegTable**	**ABC-ES**	**ABCx+ADVM-m5**	**MABC**	**ABC+A-DVM**	**ABCx-m5**	**GBESTABC+A-DVM**	**EPSO**	**ABC**	**ABCx-m1**	**GBESTABC**	**GBESTABC2+A-DVM**	**GBESTABC2**	**ABCx+ADVM-m1**	**MABC+ADVM**
PSO	1.88668 × 10${}^{-10}$	6.15459 × 10${}^{-5}$	0.00988418	8.29814 × 10${}^{-6}$	0.000401945	0.0738762	0.125063	4.85361 × 10${}^{-7}$	0.000186338	0.181179	0.0507516	0.055547	0.000186338	0.00988418
ABC-ES	-	7.0328 × 10${}^{-10}$	2.59815 × 10${}^{-12}$	9.38541 × 10${}^{-12}$	2.78175 × 10${}^{-10}$	1.19757 × 10${}^{-11}$	1.37041 × 10${}^{-11}$	1.10665 × 10${}^{-11}$	6.43181 × 10${}^{-10}$	1.17704 × 10${}^{-11}$	6.81594 × 10${}^{-12}$	8.24062 × 10${}^{-12}$	8.46924 × 10${}^{-10}$	2.59815 × 10${}^{-12}$
ABCx+ADVM-m5	-	-	6.66123 × 10${}^{-11}$	3.34893 × 10${}^{-11}$	0.432483	2.41841 × 10${}^{-9}$	8.32168 × 10${}^{-9}$	2.57089 × 10${}^{-11}$	0.0611635	4.60855 × 10${}^{-9}$	5.24118 × 10${}^{-10}$	7.24385 × 10${}^{-10}$	0.0768193	6.66123 × 10${}^{-11}$
MABC	-	-	-	2.00663 × 10${}^{-5}$	8.45378 × 10${}^{-10}$	0.175828	0.153162	2.20242 × 10${}^{-7}$	2.60004 × 10${}^{-12}$	0.0467708	0.146779	0.154137	2.60004 × 10${}^{-12}$	0.18406
ABC+A-DVM	-	-	-	-	5.55389 × 10${}^{-10}$	0.00258024	0.00551008	0.0612246	9.39154 × 10${}^{-12}$	0.00014159	2.62516 × 10${}^{-5}$	5.04706 × 10${}^{-5}$	9.39154 × 10${}^{-12}$	2.00663 × 10${}^{-5}$
ABCx-m5	-	-	-	-	-	5.96732 × 10${}^{-9}$	1.26859 × 10${}^{-8}$	4.07016 × 10${}^{-10}$	0.119918	9.61915 × 10${}^{-9}$	2.85227 × 10${}^{-9}$	3.41938 × 10${}^{-9}$	0.132156	8.45378 × 10${}^{-10}$
GBESTABC+A-DVM	-	-	-	-	-	-	0.308042	0.000411954	1.19833 × 10${}^{-11}$	0.253375	0.396563	0.421904	1.19833 × 10${}^{-11}$	0.175828
EPSO	-	-	-	-	-	-	-	0.0012239	5.50656 × 10${}^{-11}$	0.48183	0.276969	0.288925	4.10246 × 10${}^{-11}$	0.153162
ABC	-	-	-	-	-	-	-	-	1.10736 × 10${}^{-11}$	1.06775 × 10${}^{-5}$	8.00576 × 10${}^{-7}$	1.62891 × 10${}^{-6}$	1.10736 × 10${}^{-11}$	2.20242 × 10${}^{-7}$
ABCx-m1	-	-	-	-	-	-	-	-	-	1.1778 × 10${}^{-11}$	6.82052 × 10${}^{-12}$	8.24607 × 10${}^{-12}$	0.159152	2.60004 × 10${}^{-12}$
GBESTABC	-	-	-	-	-	-	-	-	-	-	0.160971	0.176564	1.1778 × 10${}^{-11}$	0.0467708
GBESTABC2+A-DVM	-	-	-	-	-	-	-	-	-	-	-	0.4831	6.82052 × 10${}^{-12}$	0.146779
GBESTABC2	-	-	-	-	-	-	-	-	-	-	-	-	8.24607 × 10${}^{-12}$	0.154137
ABCx+ADVM-m1	-	-	-	-	-	-	-	-	-	-	-	-	-	2.60004 × 10${}^{-12}$
**CrownedCross**	**ABC-ES**	**ABCx+ADVM-m5**	**MABC**	**ABC+A-DVM**	**ABCx-m5**	**GBESTABC+A-DVM**	**EPSO**	**ABC**	**ABCx-m1**	**GBESTABC**	**GBESTABC2+A-DVM**	**GBESTABC2**	**ABCx+ADVM-m1**	**MABC+ADVM**
PSO	1.88774 × 10${}^{-10}$	6.15459 × 10${}^{-5}$	0.00313874	2.16704 × 10${}^{-5}$	0.000401945	0.0308299	0.125063	1.24881 × 10${}^{-5}$	0.000186338	0.225414	0.0131181	0.156157	0.000186267	0.00313874
ABC-ES	-	7.03648 × 10${}^{-10}$	3.88432 × 10${}^{-12}$	8.46104 × 10${}^{-12}$	2.78328 × 10${}^{-10}$	1.2895 × 10${}^{-11}$	1.37128 × 10${}^{-11}$	9.49637 × 10${}^{-12}$	1.74856 × 10${}^{-9}$	1.19987 × 10${}^{-11}$	1.01877 × 10${}^{-11}$	7.7844 × 10${}^{-12}$	3.68616 × 10${}^{-10}$	3.88432 × 10${}^{-12}$
ABCx+ADVM-m5	-	-	7.59201 × 10${}^{-11}$	3.5606 × 10${}^{-11}$	0.432483	1.76096 × 10${}^{-9}$	8.32168 × 10${}^{-9}$	3.66889 × 10${}^{-11}$	0.135336	5.53236 × 10${}^{-9}$	5.39866 × 10${}^{-10}$	1.61048 × 10${}^{-9}$	0.0225627	7.59201 × 10${}^{-11}$
MABC	-	-	-	0.00272637	1.01263 × 10${}^{-9}$	0.391053	0.108087	0.00110036	3.88432 × 10${}^{-12}$	0.0149546	0.414888	0.00657923	3.87885 × 10${}^{-12}$	0.18406
ABC+A-DVM	-	-	-	-	5.93316 × 10${}^{-10}$	0.0257818	0.00865908	0.338518	8.46104 × 10${}^{-12}$	8.60446 × 10${}^{-5}$	0.0107089	7.74196 × 10${}^{-6}$	8.44989 × 10${}^{-12}$	0.00272637
ABCx-m5	-	-	-	-	-	7.12191 × 10${}^{-9}$	1.26859 × 10${}^{-8}$	6.34878 × 10${}^{-10}$	0.27961	1.49781 × 10${}^{-8}$	3.31439 × 10${}^{-9}$	6.25234 × 10${}^{-9}$	0.0374054	1.01263 × 10${}^{-9}$
GBESTABC+A-DVM	-	-	-	-	-	-	0.273051	0.0170544	1.2895 × 10${}^{-11}$	0.119454	0.432415	0.141106	1.28786 × 10${}^{-11}$	0.391053
EPSO	-	-	-	-	-	-	-	0.0072814	6.07163 × 10${}^{-11}$	0.375122	0.173769	0.5	8.94474 × 10${}^{-11}$	0.108087
ABC	-	-	-	-	-	-	-	-	9.49637 × 10${}^{-12}$	4.65081 × 10${}^{-5}$	0.00577667	3.90672 × 10${}^{-6}$	9.48398 × 10${}^{-12}$	0.00110036
ABCx-m1	-	-	-	-	-	-	-	-	-	1.19987 × 10${}^{-11}$	1.01877 × 10${}^{-11}$	7.7844 × 10${}^{-12}$	0.00636482	3.88432 × 10${}^{-12}$
GBESTABC	-	-	-	-	-	-	-	-	-	-	0.0472774	0.351055	1.19833 × 10${}^{-11}$	0.0149546
GBESTABC2+A-DVM	-	-	-	-	-	-	-	-	-	-	-	0.0482569	1.01745 × 10${}^{-11}$	0.414888
GBESTABC2	-	-	-	-	-	-	-	-	-	-	-	-	7.77407 × 10${}^{-12}$	0.00657923
ABCx+ADVM-m1	-	-	-	-	-	-	-	-	-	-	-	-	-	3.87885 × 10${}^{-12}$

**Table 3:** Pairwise U-test analysis from Damavandi to Rastrigin functions.

Damavandi	ABC-ES	ABCx+ADVM-m5	MABC	ABC+A-DVM	ABCx-m5	GBESTABC+A-DVM	EPSO	ABC	ABCx-m1	GBESTABC	GBESTABC2+A-DVM	GBESTABC2	ABCx+ADVM-m1	MABC+ADVM
PSO	5.82529 × 10${}^{-14}$	0.18406	0.00551754	0.00551754	0.18406	0.0804011	0.0054458	0.0804011	0.18406	0.166855	0.0407615	0.0407615	0.166855	0.00551754
ABC-ES	-	5.82529 × 10${}^{-14}$	7.92983 × 10${}^{-13}$	7.92983 × 10${}^{-13}$	5.82529 × 10${}^{-14}$	1.63123 × 10${}^{-13}$	1.23252 × 10${}^{-9}$	1.63123 × 10${}^{-13}$	5.82529 × 10${}^{-14}$	9.97923 × 10${}^{-14}$	2.55617 × 10${}^{-13}$	2.55617 × 10${}^{-13}$	4.01815 × 10${}^{-13}$	7.92983 × 10${}^{-13}$
ABCx+ADVM-m5	-	-	0.00551754	0.00551754	0.18406	0.0804011	0.0054458	0.0804011	0.18406	0.166855	0.0407615	0.0407615	0.166855	0.00551754
MABC	-	-	-	0.453671	0.00551754	0.0903332	0.355428	0.0682646	0.00551754	0.0289726	0.119396	0.199101	0.0289726	0.18406
ABC+A-DVM	-	-	-	-	0.00551754	0.0903332	0.355428	0.0650302	0.00551754	0.0289726	0.119396	0.199101	0.0289726	0.453671
ABCx-m5	-	-	-	-	-	0.0804011	0.0054458	0.0804011	0.18406	0.166855	0.0407615	0.0407615	0.166855	0.00551754
GBESTABC+A-DVM	-	-	-	-	-	-	0.0503713	0.47955	0.0804011	0.298595	0.361414	0.29469	0.298595	0.0903332
EPSO	-	-	-	-	-	-	-	0.0503713	0.0054458	0.0197735	0.101188	0.101188	0.0238162	0.355428
ABC	-	-	-	-	-	-	-	-	0.0804011	0.298595	0.349924	0.29469	0.298595	0.0682646
ABCx-m1	-	-	-	-	-	-	-	-	-	0.166855	0.0407615	0.0407615	0.166855	0.00551754
GBESTABC	-	-	-	-	-	-	-	-	-	-	0.169209	0.152523	0.5	0.0289726
GBESTABC2+A-DVM	-	-	-	-	-	-	-	-	-	-	-	0.454781	0.169209	0.119396
GBESTABC2	-	-	-	-	-	-	-	-	-	-	-	-	0.169209	0.199101
ABCx+ADVM-m1	-	-	-	-	-	-	-	-	-	-	-	-	-	0.0289726
**DeVilliersGlasser02**	**ABC-ES**	**ABCx+ADVM-m5**	**MABC**	**ABC+A-DVM**	**ABCx-m5**	**GBESTABC+A-DVM**	**EPSO**	**ABC**	**ABCx-m1**	**GBESTABC**	**GBESTABC2+A-DVM**	**GBESTABC2**	**ABCx+ADVM-m1**	**MABC+ADVM**
PSO	1.50993 × 10${}^{-11}$	1.50993 × 10${}^{-11}$	1.50993 × 10${}^{-11}$	6.0589 × 10${}^{-13}$	1.50993 × 10${}^{-11}$	8.60126 × 10${}^{-13}$	1.66919 × 10${}^{-11}$	6.0589 × 10${}^{-13}$	1.50993 × 10${}^{-11}$	6.0589 × 10${}^{-13}$	6.0589 × 10${}^{-13}$	6.0589 × 10${}^{-13}$	1.50993 × 10${}^{-11}$	1.50993 × 10${}^{-11}$
ABC-ES	-	4.87775 × 10${}^{-10}$	1.50993 × 10${}^{-11}$	6.0589 × 10${}^{-13}$	2.09984 × 10${}^{-10}$	8.60126 × 10${}^{-13}$	1.50993 × 10${}^{-11}$	6.0589 × 10${}^{-13}$	0.0151587	6.0589 × 10${}^{-13}$	6.0589 × 10${}^{-13}$	6.0589 × 10${}^{-13}$	0.152088	1.50993 × 10${}^{-11}$
ABCx+ADVM-m5	-	-	1.50993 × 10${}^{-11}$	6.0589 × 10${}^{-13}$	0.245891	8.60126 × 10${}^{-13}$	1.50993 × 10${}^{-11}$	6.0589 × 10${}^{-13}$	3.82939 × 10${}^{-5}$	6.0589 × 10${}^{-13}$	6.0589 × 10${}^{-13}$	6.0589 × 10${}^{-13}$	2.05888 × 10${}^{-6}$	1.50993 × 10${}^{-11}$
MABC	-	-	-	6.0589 × 10${}^{-13}$	1.50993 × 10${}^{-11}$	8.60126 × 10${}^{-13}$	5.46835 × 10${}^{-11}$	6.0589 × 10${}^{-13}$	1.50993 × 10${}^{-11}$	6.0589 × 10${}^{-13}$	6.0589 × 10${}^{-13}$	6.0589 × 10${}^{-13}$	2.48758 × 10${}^{-11}$	0.18406
ABC+A-DVM	-	-	-	-	6.0589 × 10${}^{-13}$	0.166855	6.0589 × 10${}^{-13}$	0.18406	6.0589 × 10${}^{-13}$	0.18406	0.18406	0.18406	6.0589 × 10${}^{-13}$	6.0589 × 10${}^{-13}$
ABCx-m5	-	-	-	-	-	8.60126 × 10${}^{-13}$	1.50993 × 10${}^{-11}$	6.0589 × 10${}^{-13}$	2.6325 × 10${}^{-5}$	6.0589 × 10${}^{-13}$	6.0589 × 10${}^{-13}$	6.0589 × 10${}^{-13}$	1.66208 × 10${}^{-6}$	1.50993 × 10${}^{-11}$
GBESTABC+A-DVM	-	-	-	-	-	-	8.60126 × 10${}^{-13}$	0.166855	1.2055 × 10${}^{-12}$	0.166855	0.166855	0.166855	1.07747 × 10${}^{-12}$	8.60126 × 10${}^{-13}$
EPSO	-	-	-	-	-	-	-	6.0589 × 10${}^{-13}$	1.50993 × 10${}^{-11}$	6.0589 × 10${}^{-13}$	6.0589 × 10${}^{-13}$	6.0589 × 10${}^{-13}$	1.50993 × 10${}^{-11}$	5.46835 × 10${}^{-11}$
ABC	-	-	-	-	-	-	-	-	6.0589 × 10${}^{-13}$	0.18406	0.18406	0.18406	6.0589 × 10${}^{-13}$	6.0589 × 10${}^{-13}$
ABCx-m1	-	-	-	-	-	-	-	-	-	6.0589 × 10${}^{-13}$	6.0589 × 10${}^{-13}$	6.0589 × 10${}^{-13}$	0.189518	1.50993 × 10${}^{-11}$
GBESTABC	-	-	-	-	-	-	-	-	-	-	0.18406	0.18406	6.0589 × 10${}^{-13}$	6.0589 × 10${}^{-13}$
GBESTABC2+A-DVM	-	-	-	-	-	-	-	-	-	-	-	0.18406	6.0589 × 10${}^{-13}$	6.0589 × 10${}^{-13}$
GBESTABC2	-	-	-	-	-	-	-	-	-	-	-	-	6.0589 × 10${}^{-13}$	6.0589 × 10${}^{-13}$
ABCx+ADVM-m1	-	-	-	-	-	-	-	-	-	-	-	-	-	2.48758 × 10${}^{-11}$
**Griewank**	**ABC-ES**	**ABCx+ADVM-m5**	**MABC**	**ABC+A-DVM**	**ABCx-m5**	**GBESTABC+A-DVM**	**EPSO**	**ABC**	**ABCx-m1**	**GBESTABC**	**GBESTABC2+A-DVM**	**GBESTABC2**	**ABCx+ADVM-m1**	**MABC+ADVM**
PSO	1.19833 × 10${}^{-11}$	1.50993 × 10${}^{-11}$	1.50709 × 10${}^{-11}$	6.0589 × 10${}^{-13}$	1.50898 × 10${}^{-11}$	6.0589 × 10${}^{-13}$	1.18284 × 10${}^{-12}$	8.60126 × 10${}^{-13}$	1.50993 × 10${}^{-11}$	8.60126 × 10${}^{-13}$	6.0589 × 10${}^{-13}$	1.18192 × 10${}^{-12}$	1.50898 × 10${}^{-11}$	1.50709 × 10${}^{-11}$
ABC-ES	-	5.81883 × 10${}^{-10}$	0.00772719	1.10591 × 10${}^{-6}$	4.39882 × 10${}^{-10}$	1.10591 × 10${}^{-6}$	0.000100603	1.25899 × 10${}^{-5}$	1.05827 × 10${}^{-10}$	3.93803 × 10${}^{-6}$	1.10591 × 10${}^{-6}$	7.56738 × 10${}^{-6}$	2.50191 × 10${}^{-10}$	0.00772719
ABCx+ADVM-m5	-	-	0.000601919	6.0589 × 10${}^{-13}$	0.41801	6.0589 × 10${}^{-13}$	6.19215 × 10${}^{-10}$	2.28091 × 10${}^{-11}$	1.73883 × 10${}^{-5}$	8.60126 × 10${}^{-13}$	6.0589 × 10${}^{-13}$	1.18192 × 10${}^{-12}$	5.4519 × 10${}^{-6}$	0.000601919
MABC	-	-	-	8.27367 × 10${}^{-12}$	0.000528437	8.27367 × 10${}^{-12}$	5.04433 × 10${}^{-9}$	2.27682 × 10${}^{-10}$	1.38985 × 10${}^{-7}$	3.32294 × 10${}^{-11}$	8.27367 × 10${}^{-12}$	2.55281 × 10${}^{-11}$	3.01539 × 10${}^{-7}$	0.18406
ABC+A-DVM	-	-	-	-	6.05396 × 10${}^{-13}$	0.18406	0.0804011	0.166855	6.0589 × 10${}^{-13}$	0.166855	0.18406	0.080371	6.05396 × 10${}^{-13}$	8.27367 × 10${}^{-12}$
ABCx-m5	-	-	-	-	-	6.05396 × 10${}^{-13}$	6.18852 × 10${}^{-10}$	2.27933 × 10${}^{-11}$	2.89174 × 10${}^{-5}$	8.59442 × 10${}^{-13}$	6.05396 × 10${}^{-13}$	1.181 × 10${}^{-12}$	7.14526 × 10${}^{-6}$	0.000528437
GBESTABC+A-DVM	-	-	-	-	-	-	0.0804011	0.166855	6.0589 × 10${}^{-13}$	0.166855	0.18406	0.080371	6.05396 × 10${}^{-13}$	8.27367 × 10${}^{-12}$
EPSO	-	-	-	-	-	-	-	0.272013	3.44112 × 10${}^{-10}$	0.272013	0.0804011	0.479548	3.79508 × 10${}^{-10}$	5.04433 × 10${}^{-9}$
ABC	-	-	-	-	-	-	-	-	3.27579 × 10${}^{-12}$	0.5	0.166855	0.298577	4.54799 × 10${}^{-12}$	2.27682 × 10${}^{-10}$
ABCx-m1	-	-	-	-	-	-	-	-	-	8.60126 × 10${}^{-13}$	6.0589 × 10${}^{-13}$	1.18192 × 10${}^{-12}$	0.353084	1.38985 × 10${}^{-7}$
GBESTABC	-	-	-	-	-	-	-	-	-	-	0.166855	0.298577	8.59442 × 10${}^{-13}$	3.32294 × 10${}^{-11}$
GBESTABC2+A-DVM	-	-	-	-	-	-	-	-	-	-	-	0.080371	6.05396 × 10${}^{-13}$	8.27367 × 10${}^{-12}$
GBESTABC2	-	-	-	-	-	-	-	-	-	-	-	-	1.181 × 10${}^{-12}$	2.55281 × 10${}^{-11}$
ABCx+ADVM-m1	-	-	-	-	-	-	-	-	-	-	-	-	-	3.01539 × 10${}^{-7}$
**Rastrigin**	**ABC-ES**	**ABCx+ADVM-m5**	**MABC**	**ABC+A-DVM**	**ABCx-m5**	**GBESTABC+A-DVM**	**EPSO**	**ABC**	**ABCx-m1**	**GBESTABC**	**GBESTABC2+A-DVM**	**GBESTABC2**	**ABCx+ADVM-m1**	**MABC+ADVM**
PSO	1.50993 × 10${}^{-11}$	1.50993 × 10${}^{-11}$	1.50993 × 10${}^{-11}$	6.0589 × 10${}^{-13}$	1.50993 × 10${}^{-11}$	8.60126 × 10${}^{-13}$	1.66919 × 10${}^{-11}$	6.0589 × 10${}^{-13}$	1.50993 × 10${}^{-11}$	6.0589 × 10${}^{-13}$	6.0589 × 10${}^{-13}$	6.0589 × 10${}^{-13}$	1.50993 × 10${}^{-11}$	1.50993 × 10${}^{-11}$
ABC-ES	-	4.87775 × 10${}^{-10}$	1.50993 × 10${}^{-11}$	6.0589 × 10${}^{-13}$	2.09984 × 10${}^{-10}$	8.60126 × 10${}^{-13}$	1.50993 × 10${}^{-11}$	6.0589 × 10${}^{-13}$	0.0151587	6.0589 × 10${}^{-13}$	6.0589 × 10${}^{-13}$	6.0589 × 10${}^{-13}$	0.152088	1.50993 × 10${}^{-11}$
ABCx+ADVM-m5	-	-	1.50993 × 10${}^{-11}$	6.0589 × 10${}^{-13}$	0.245891	8.60126 × 10${}^{-13}$	1.50993 × 10${}^{-11}$	6.0589 × 10${}^{-13}$	3.82939 × 10${}^{-5}$	6.0589 × 10${}^{-13}$	6.0589 × 10${}^{-13}$	6.0589 × 10${}^{-13}$	2.05888 × 10${}^{-6}$	1.50993 × 10${}^{-11}$
MABC	-	-	-	6.0589 × 10${}^{-13}$	1.50993 × 10${}^{-11}$	8.60126 × 10${}^{-13}$	5.46835 × 10${}^{-11}$	6.0589 × 10${}^{-13}$	1.50993 × 10${}^{-11}$	6.0589 × 10${}^{-13}$	6.0589 × 10${}^{-13}$	6.0589 × 10${}^{-13}$	2.48758 × 10${}^{-11}$	0.18406
ABC+A-DVM	-	-	-	-	6.0589 × 10${}^{-13}$	0.166855	6.0589 × 10${}^{-13}$	0.18406	6.0589 × 10${}^{-13}$	0.18406	0.18406	0.18406	6.0589 × 10${}^{-13}$	6.0589 × 10${}^{-13}$
ABCx-m5	-	-	-	-	-	8.60126 × 10${}^{-13}$	1.50993 × 10${}^{-11}$	6.0589 × 10${}^{-13}$	2.6325 × 10${}^{-5}$	6.0589 × 10${}^{-13}$	6.0589 × 10${}^{-13}$	6.0589 × 10${}^{-13}$	1.66208 × 10${}^{-6}$	1.50993 × 10${}^{-11}$
GBESTABC+A-DVM	-	-	-	-	-	-	8.60126 × 10${}^{-13}$	0.166855	1.2055 × 10${}^{-12}$	0.166855	0.166855	0.166855	1.07747 × 10${}^{-12}$	8.60126 × 10${}^{-13}$
EPSO	-	-	-	-	-	-	-	6.0589 × 10${}^{-13}$	1.50993 × 10${}^{-11}$	6.0589 × 10${}^{-13}$	6.0589 × 10${}^{-13}$	6.0589 × 10${}^{-13}$	1.50993 × 10${}^{-11}$	5.46835 × 10${}^{-11}$
ABC	-	-	-	-	-	-	-	-	6.0589 × 10${}^{-13}$	0.18406	0.18406	0.18406	6.0589 × 10${}^{-13}$	6.0589 × 10${}^{-13}$
ABCx-m1	-	-	-	-	-	-	-	-	-	6.0589 × 10${}^{-13}$	6.0589 × 10${}^{-13}$	6.0589 × 10${}^{-13}$	0.189518	1.50993 × 10${}^{-11}$
GBESTABC	-	-	-	-	-	-	-	-	-	-	0.18406	0.18406	6.0589 × 10${}^{-13}$	6.0589 × 10${}^{-13}$
GBESTABC2+A-DVM	-	-	-	-	-	-	-	-	-	-	-	0.18406	6.0589 × 10${}^{-13}$	6.0589 × 10${}^{-13}$
GBESTABC2	-	-	-	-	-	-	-	-	-	-	-	-	6.0589 × 10${}^{-13}$	6.0589 × 10${}^{-13}$
ABCx+ADVM-m1	-	-	-	-	-	-	-	-	-	-	-	-	-	2.48758 × 10${}^{-11}$

**Table 4:** Pairwise U-test analysis from Rosenbrock to Trefethen functions.

Rosenbrock	ABC-ES	ABCx+ADVM-m5	MABC	ABC+A-DVM	ABCx-m5	GBESTABC+A-DVM	EPSO	ABC	ABCx-m1	GBESTABC	GBESTABC2+A-DVM	GBESTABC2	ABCx+ADVM-m1	MABC+ADVM
PSO	1.50993 × 10${}^{-11}$	1.50993 × 10${}^{-11}$	1.50993 × 10${}^{-11}$	6.0589 × 10${}^{-13}$	1.50993 × 10${}^{-11}$	8.60126 × 10${}^{-13}$	1.66919 × 10${}^{-11}$	6.0589 × 10${}^{-13}$	1.50993 × 10${}^{-11}$	6.0589 × 10${}^{-13}$	6.0589 × 10${}^{-13}$	6.0589 × 10${}^{-13}$	1.50993 × 10${}^{-11}$	1.50993 × 10${}^{-11}$
ABC-ES	-	4.87775 × 10${}^{-10}$	1.50993 × 10${}^{-11}$	6.0589 × 10${}^{-13}$	2.09984 × 10${}^{-10}$	8.60126 × 10${}^{-13}$	1.50993 × 10${}^{-11}$	6.0589 × 10${}^{-13}$	0.0151587	6.0589 × 10${}^{-13}$	6.0589 × 10${}^{-13}$	6.0589 × 10${}^{-13}$	0.152088	1.50993 × 10${}^{-11}$
ABCx+ADVM-m5	-	-	1.50993 × 10${}^{-11}$	6.0589 × 10${}^{-13}$	0.245891	8.60126 × 10${}^{-13}$	1.50993 × 10${}^{-11}$	6.0589 × 10${}^{-13}$	3.82939 × 10${}^{-5}$	6.0589 × 10${}^{-13}$	6.0589 × 10${}^{-13}$	6.0589 × 10${}^{-13}$	2.05888 × 10${}^{-6}$	1.50993 × 10${}^{-11}$
MABC	-	-	-	6.0589 × 10${}^{-13}$	1.50993 × 10${}^{-11}$	8.60126 × 10${}^{-13}$	5.46835 × 10${}^{-11}$	6.0589 × 10${}^{-13}$	1.50993 × 10${}^{-11}$	6.0589 × 10${}^{-13}$	6.0589 × 10${}^{-13}$	6.0589 × 10${}^{-13}$	2.48758 × 10${}^{-11}$	0.18406
ABC+A-DVM	-	-	-	-	6.0589 × 10${}^{-13}$	0.166855	6.0589 × 10${}^{-13}$	0.18406	6.0589 × 10${}^{-13}$	0.18406	0.18406	0.18406	6.0589 × 10${}^{-13}$	6.0589 × 10${}^{-13}$
ABCx-m5	-	-	-	-	-	8.60126 × 10${}^{-13}$	1.50993 × 10${}^{-11}$	6.0589 × 10${}^{-13}$	2.6325 × 10${}^{-5}$	6.0589 × 10${}^{-13}$	6.0589 × 10${}^{-13}$	6.0589 × 10${}^{-13}$	1.66208 × 10${}^{-6}$	1.50993 × 10${}^{-11}$
GBESTABC+A-DVM	-	-	-	-	-	-	8.60126 × 10${}^{-13}$	0.166855	1.2055 × 10${}^{-12}$	0.166855	0.166855	0.166855	1.07747 × 10${}^{-12}$	8.60126 × 10${}^{-13}$
EPSO	-	-	-	-	-	-	-	6.0589 × 10${}^{-13}$	1.50993 × 10${}^{-11}$	6.0589 × 10${}^{-13}$	6.0589 × 10${}^{-13}$	6.0589 × 10${}^{-13}$	1.50993 × 10${}^{-11}$	5.46835 × 10${}^{-11}$
ABC	-	-	-	-	-	-	-	-	6.0589 × 10${}^{-13}$	0.18406	0.18406	0.18406	6.0589 × 10${}^{-13}$	6.0589 × 10${}^{-13}$
ABCx-m1	-	-	-	-	-	-	-	-	-	6.0589 × 10${}^{-13}$	6.0589 × 10${}^{-13}$	6.0589 × 10${}^{-13}$	0.189518	1.50993 × 10${}^{-11}$
GBESTABC	-	-	-	-	-	-	-	-	-	-	0.18406	0.18406	6.0589 × 10${}^{-13}$	6.0589 × 10${}^{-13}$
GBESTABC2+A-DVM	-	-	-	-	-	-	-	-	-	-	-	0.18406	6.0589 × 10${}^{-13}$	6.0589 × 10${}^{-13}$
GBESTABC2	-	-	-	-	-	-	-	-	-	-	-	-	6.0589 × 10${}^{-13}$	6.0589 × 10${}^{-13}$
ABCx+ADVM-m1	-	-	-	-	-	-	-	-	-	-	-	-	-	2.48758 × 10${}^{-11}$
**Schwefel** **06**	**ABC-ES**	**ABCx+ADVM-m5**	**MABC**	**ABC+A-DVM**	**ABCx-m5**	**GBESTABC+A-DVM**	**EPSO**	**ABC**	**ABCx-m1**	**GBESTABC**	**GBESTABC2+A-DVM**	**GBESTABC2**	**ABCx+ADVM-m1**	**MABC+ADVM**
PSO	0.0013875	6.90317 × 10${}^{-12}$	6.0589 × 10${}^{-13}$	6.0589 × 10${}^{-13}$	2.1558 × 10${}^{-11}$	8.27367 × 10${}^{-12}$	0.18406	6.0589 × 10${}^{-13}$	0.18406	9.6525 × 10${}^{-11}$	6.05396 × 10${}^{-13}$	6.0589 × 10${}^{-13}$	0.18406	6.0589 × 10${}^{-13}$
ABC-ES	-	1.46217 × 10${}^{-7}$	4.66114 × 10${}^{-12}$	4.66114 × 10${}^{-12}$	1.83084 × 10${}^{-6}$	1.75567 × 10${}^{-10}$	0.0013875	4.66114 × 10${}^{-12}$	0.0013875	7.12005 × 10${}^{-9}$	4.65791 × 10${}^{-12}$	4.66114 × 10${}^{-12}$	0.0013875	4.66114 × 10${}^{-12}$
ABCx+ADVM-m5	-	-	1.29772 × 10${}^{-11}$	1.29772 × 10${}^{-11}$	0.120463	8.41872 × 10${}^{-8}$	6.90317 × 10${}^{-12}$	1.29772 × 10${}^{-11}$	6.90317 × 10${}^{-12}$	1.50954 × 10${}^{-5}$	1.2969 × 10${}^{-11}$	1.29772 × 10${}^{-11}$	6.90317 × 10${}^{-12}$	1.29772 × 10${}^{-11}$
MABC	-	-	-	0.100474	1.17328 × 10${}^{-11}$	1.50709 × 10${}^{-11}$	6.0589 × 10${}^{-13}$	0.0496288	6.0589 × 10${}^{-13}$	1.49767 × 10${}^{-11}$	3.05885 × 10${}^{-10}$	1.59837 × 10${}^{-9}$	6.0589 × 10${}^{-13}$	0.18406
ABC+A-DVM	-	-	-	-	1.17328 × 10${}^{-11}$	1.50709 × 10${}^{-11}$	6.0589 × 10${}^{-13}$	0.294726	6.0589 × 10${}^{-13}$	1.6557 × 10${}^{-11}$	1.53985 × 10${}^{-8}$	5.78327 × 10${}^{-8}$	6.0589 × 10${}^{-13}$	0.100474
ABCx-m5	-	-	-	-	-	1.57018 × 10${}^{-8}$	2.1558 × 10${}^{-11}$	1.17328 × 10${}^{-11}$	2.1558 × 10${}^{-11}$	3.47585 × 10${}^{-6}$	1.17253 × 10${}^{-11}$	1.17328 × 10${}^{-11}$	2.1558 × 10${}^{-11}$	1.17328 × 10${}^{-11}$
GBESTABC+A-DVM	-	-	-	-	-	-	8.27367 × 10${}^{-12}$	1.84143 × 10${}^{-11}$	8.27367 × 10${}^{-12}$	0.160786	2.41277 × 10${}^{-10}$	2.248 × 10${}^{-11}$	8.27367 × 10${}^{-12}$	1.50709 × 10${}^{-11}$
EPSO	-	-	-	-	-	-	-	6.0589 × 10${}^{-13}$	0.18406	9.6525 × 10${}^{-11}$	6.05396 × 10${}^{-13}$	6.0589 × 10${}^{-13}$	0.18406	6.0589 × 10${}^{-13}$
ABC	-	-	-	-	-	-	-	-	6.0589 × 10${}^{-13}$	2.7253 × 10${}^{-11}$	1.01375 × 10${}^{-7}$	7.45902 × 10${}^{-7}$	6.0589 × 10${}^{-13}$	0.0496288
ABCx-m1	-	-	-	-	-	-	-	-	-	9.6525 × 10${}^{-11}$	6.05396 × 10${}^{-13}$	6.0589 × 10${}^{-13}$	0.18406	6.0589 × 10${}^{-13}$
GBESTABC	-	-	-	-	-	-	-	-	-	-	5.82549 × 10${}^{-10}$	1.0692 × 10${}^{-10}$	9.6525 × 10${}^{-11}$	1.49767 × 10${}^{-11}$
GBESTABC2+A-DVM	-	-	-	-	-	-	-	-	-	-	-	0.17966	6.05396 × 10${}^{-13}$	3.05885 × 10${}^{-10}$
GBESTABC2	-	-	-	-	-	-	-	-	-	-	-	-	6.0589 × 10${}^{-13}$	1.59837 × 10${}^{-9}$
ABCx+ADVM-m1	-	-	-	-	-	-	-	-	-	-	-	-	-	6.0589 × 10${}^{-13}$
**SineEnvelope**	**ABC-ES**	**ABCx+ADVM-m5**	**MABC**	**ABC+A-DVM**	**ABCx-m5**	**GBESTABC+A-DVM**	**EPSO**	**ABC**	**ABCx-m1**	**GBESTABC**	**GBESTABC2+A-DVM**	**GBESTABC2**	**ABCx+ADVM-m1**	**MABC+ADVM**
PSO	1.50993 × 10${}^{-11}$	1.50993 × 10${}^{-11}$	1.50993 × 10${}^{-11}$	1.50993 × 10${}^{-11}$	1.50993 × 10${}^{-11}$	1.50993 × 10${}^{-11}$	3.69454 × 10${}^{-11}$	1.50993 × 10${}^{-11}$	1.50993 × 10${}^{-11}$	1.50993 × 10${}^{-11}$	1.50993 × 10${}^{-11}$	1.50993 × 10${}^{-11}$	1.50993 × 10${}^{-11}$	1.50993 × 10${}^{-11}$
ABC-ES	-	3.06052 × 10${}^{-10}$	1.07702 × 10${}^{-6}$	1.66919 × 10${}^{-11}$	3.06052 × 10${}^{-10}$	1.50993 × 10${}^{-11}$	1.50993 × 10${}^{-11}$	1.50993 × 10${}^{-11}$	3.26307 × 10${}^{-7}$	1.50993 × 10${}^{-11}$	1.50993 × 10${}^{-11}$	1.50993 × 10${}^{-11}$	2.4713 × 10${}^{-5}$	1.07702 × 10${}^{-6}$
ABCx+ADVM-m5	-	-	2.03858 × 10${}^{-11}$	1.09737 × 10${}^{-8}$	0.380914	5.46835 × 10${}^{-11}$	1.50993 × 10${}^{-11}$	1.11363 × 10${}^{-9}$	0.461721	8.06613 × 10${}^{-11}$	4.07637 × 10${}^{-11}$	1.30493 × 10${}^{-10}$	0.12594	2.03858 × 10${}^{-11}$
MABC	-	-	-	1.50993 × 10${}^{-11}$	2.48758 × 10${}^{-11}$	1.50993 × 10${}^{-11}$	1.50993 × 10${}^{-11}$	1.50993 × 10${}^{-11}$	1.57944 × 10${}^{-10}$	1.50993 × 10${}^{-11}$	1.50993 × 10${}^{-11}$	1.50993 × 10${}^{-11}$	1.91245 × 10${}^{-9}$	0.18406
ABC+A-DVM	-	-	-	-	7.79038 × 10${}^{-9}$	0.00348622	1.50993 × 10${}^{-11}$	0.369699	5.45343 × 10${}^{-6}$	0.00131214	0.210193	0.173914	1.09794 × 10${}^{-7}$	1.50993 × 10${}^{-11}$
ABCx-m5	-	-	-	-	-	4.95931 × 10${}^{-11}$	1.50993 × 10${}^{-11}$	9.28367 × 10${}^{-10}$	0.479366	5.46835 × 10${}^{-11}$	5.46835 × 10${}^{-11}$	1.57944 × 10${}^{-10}$	0.132163	2.48758 × 10${}^{-11}$
GBESTABC+A-DVM	-	-	-	-	-	-	1.50993 × 10${}^{-11}$	0.00434219	2.34282 × 10${}^{-8}$	0.467596	0.00348622	0.0362228	4.87775 × 10${}^{-10}$	1.50993 × 10${}^{-11}$
EPSO	-	-	-	-	-	-	-	1.50993 × 10${}^{-11}$	1.50993 × 10${}^{-11}$	1.50993 × 10${}^{-11}$	1.50993 × 10${}^{-11}$	1.50993 × 10${}^{-11}$	1.50993 × 10${}^{-11}$	1.50993 × 10${}^{-11}$
ABC	-	-	-	-	-	-	-	-	3.36811 × 10${}^{-6}$	0.00211296	0.342161	0.274663	2.5461 × 10${}^{-8}$	1.50993 × 10${}^{-11}$
ABCx-m1	-	-	-	-	-	-	-	-	-	1.19487 × 10${}^{-8}$	5.13867 × 10${}^{-7}$	3.52149 × 10${}^{-7}$	0.159152	1.57944 × 10${}^{-10}$
GBESTABC	-	-	-	-	-	-	-	-	-	-	0.00159148	0.0146027	5.35089 × 10${}^{-10}$	1.50993 × 10${}^{-11}$
GBESTABC2+A-DVM	-	-	-	-	-	-	-	-	-	-	-	0.420901	3.25914 × 10${}^{-9}$	1.50993 × 10${}^{-11}$
GBESTABC2	-	-	-	-	-	-	-	-	-	-	-	-	6.55552 × 10${}^{-9}$	1.50993 × 10${}^{-11}$
ABCx+ADVM-m1	-	-	-	-	-	-	-	-	-	-	-	-	-	1.91245 × 10${}^{-9}$
**Trefethen**	**ABC-ES**	**ABCx+ADVM-m5**	**MABC**	**ABC+A-DVM**	**ABCx-m5**	**GBESTABC+A-DVM**	**EPSO**	**ABC**	**ABCx-m1**	**GBESTABC**	**GBESTABC2+A-DVM**	**GBESTABC2**	**ABCx+ADVM-m1**	**MABC+ADVM**
PSO	1.68597 × 10${}^{-7}$	4.4196 × 10${}^{-6}$	1.68597 × 10${}^{-7}$	1.68597 × 10${}^{-7}$	0.000911263	0.0473405	0.000372173	1.68597 × 10${}^{-7}$	0.0207284	0.0280822	1.68597 × 10${}^{-7}$	1.68597 × 10${}^{-7}$	0.00163145	1.68597 × 10${}^{-7}$
ABC-ES	-	0.0407021	0.18406	0.18406	0.000669594	3.19329 × 10${}^{-5}$	0.00136379	0.18406	2.23578 × 10${}^{-6}$	3.17451 × 10${}^{-5}$	0.18406	0.18406	0.000678539	0.18406
ABCx+ADVM-m5	-	-	0.0407021	0.0407021	0.0246025	0.00104272	0.0471179	0.0407021	0.000149753	0.00135916	0.0407021	0.0407021	0.0213792	0.0407021
MABC	-	-	-	0.18406	0.000669594	3.19329 × 10${}^{-5}$	0.00136379	0.18406	2.23578 × 10${}^{-6}$	3.17451 × 10${}^{-5}$	0.18406	0.18406	0.000678539	0.18406
ABC+A-DVM	-	-	-	-	0.000669594	3.19329 × 10${}^{-5}$	0.00136379	0.18406	2.23578 × 10${}^{-6}$	3.17451 × 10${}^{-5}$	0.18406	0.18406	0.000678539	0.18406
ABCx-m5	-	-	-	-	-	0.0749114	0.374724	0.000669594	0.0438242	0.109604	0.000669594	0.000669594	0.445246	0.000669594
GBESTABC+A-DVM	-	-	-	-	-	-	0.0450536	3.19329 × 10${}^{-5}$	0.474152	0.411891	3.19329 × 10${}^{-5}$	3.19329 × 10${}^{-5}$	0.0982305	3.19329 × 10${}^{-5}$
EPSO	-	-	-	-	-	-	-	0.00136379	0.0184559	0.0623302	0.00136379	0.00136379	0.326643	0.00136379
ABC	-	-	-	-	-	-	-	-	2.23578 × 10${}^{-6}$	3.17451 × 10${}^{-5}$	0.18406	0.18406	0.000678539	0.18406
ABCx-m1	-	-	-	-	-	-	-	-	-	0.375015	2.23578 × 10${}^{-6}$	2.23578 × 10${}^{-6}$	0.0753994	2.23578 × 10${}^{-6}$
GBESTABC	-	-	-	-	-	-	-	-	-	-	3.17451 × 10${}^{-5}$	3.17451 × 10${}^{-5}$	0.144437	3.17451 × 10${}^{-5}$
GBESTABC2+A-DVM	-	-	-	-	-	-	-	-	-	-	-	0.18406	0.000678539	0.18406
GBESTABC2	-	-	-	-	-	-	-	-	-	-	-	-	0.000678539	0.18406
ABCx+ADVM-m1	-	-	-	-	-	-	-	-	-	-	-	-	-	0.000678539

**Table 5:** Pairwise U-test analysis from Whitley to Zimmerman functions.

Whitley	ABC-ES	ABCx+ADVM-m5	MABC	ABC+A-DVM	ABCx-m5	GBESTABC+A-DVM	EPSO	ABC	ABCx-m1	GBESTABC	GBESTABC2+A-DVM	GBESTABC2	ABCx+ADVM-m1	MABC+ADVM
PSO	1.50993 × 10${}^{-11}$	1.50993 × 10${}^{-11}$	1.50993 × 10${}^{-11}$	1.50993 × 10${}^{-11}$	1.50993 × 10${}^{-11}$	1.50993 × 10${}^{-11}$	1.50993 × 10${}^{-11}$	1.50993 × 10${}^{-11}$	1.50993 × 10${}^{-11}$	1.50993 × 10${}^{-11}$	1.50993 × 10${}^{-11}$	1.50993 × 10${}^{-11}$	1.50993 × 10${}^{-11}$	1.50993 × 10${}^{-11}$
ABC-ES	-	7.14708 × 10${}^{-9}$	2.34282 × 10${}^{-8}$	4.24239 × 10${}^{-9}$	2.09125 × 10${}^{-9}$	2.04198 × 10${}^{-5}$	1.50993 × 10${}^{-11}$	2.53616 × 10${}^{-10}$	0.000405999	1.59837 × 10${}^{-9}$	3.88627 × 10${}^{-9}$	4.42055 × 10${}^{-7}$	3.1414 × 10${}^{-6}$	2.34282 × 10${}^{-8}$
ABCx+ADVM-m5	-	-	2.98528 × 10${}^{-5}$	4.60563 × 10${}^{-5}$	0.397923	0.000199405	1.50993 × 10${}^{-11}$	2.17654 × 10${}^{-5}$	0.467596	2.21025 × 10${}^{-6}$	1.90263 × 10${}^{-7}$	0.000119424	0.00720609	2.98528 × 10${}^{-5}$
MABC	-	-	-	0.299845	2.4713 × 10${}^{-5}$	0.205955	1.50993 × 10${}^{-11}$	0.331367	7.92305 × 10${}^{-5}$	0.105781	0.201769	0.116994	0.00515734	0.18406
ABC+A-DVM	-	-	-	-	6.62476 × 10${}^{-5}$	0.369699	1.50993 × 10${}^{-11}$	0.485258	1.68407 × 10${}^{-5}$	0.205955	0.497051	0.210193	0.000249091	0.299845
ABCx-m5	-	-	-	-	-	0.000199405	1.50993 × 10${}^{-11}$	3.17802 × 10${}^{-5}$	0.409373	2.73102 × 10${}^{-6}$	1.62777 × 10${}^{-7}$	0.000126529	0.00538131	2.4713 × 10${}^{-5}$
GBESTABC+A-DVM	-	-	-	-	-	-	1.50993 × 10${}^{-11}$	0.39223	4.89586 × 10${}^{-5}$	0.315438	0.218821	0.245891	0.000168395	0.205955
EPSO	-	-	-	-	-	-	-	1.50993 × 10${}^{-11}$	1.50993 × 10${}^{-11}$	1.50993 × 10${}^{-11}$	1.50993 × 10${}^{-11}$	1.50993 × 10${}^{-11}$	1.50993 × 10${}^{-11}$	1.50993 × 10${}^{-11}$
ABC	-	-	-	-	-	-	-	-	1.68407 × 10${}^{-5}$	0.22321	0.485258	0.236673	0.000883282	0.331367
ABCx-m1	-	-	-	-	-	-	-	-	-	2.21025 × 10${}^{-6}$	2.59284 × 10${}^{-7}$	1.29868 × 10${}^{-5}$	0.0328356	7.92305 × 10${}^{-5}$
GBESTABC	-	-	-	-	-	-	-	-	-	-	0.145236	0.479366	2.98528 × 10${}^{-5}$	0.105781
GBESTABC2+A-DVM	-	-	-	-	-	-	-	-	-	-	-	0.0953652	7.14918 × 10${}^{-6}$	0.201769
GBESTABC2	-	-	-	-	-	-	-	-	-	-	-	-	3.59939 × 10${}^{-5}$	0.116994
ABCx+ADVM-m1	-	-	-	-	-	-	-	-	-	-	-	-	-	0.00515734
**XinSheYang06**	**ABC-ES**	**ABCx+ADVM-m5**	**MABC**	**ABC+A-DVM**	**ABCx-m5**	**GBESTABC+A-DVM**	**EPSO**	**ABC**	**ABCx-m1**	**GBESTABC**	**GBESTABC2+A-DVM**	**GBESTABC2**	**ABCx+ADVM-m1**	**MABC+ADVM**
PSO	0.0407021	0.18406	0.18406	0.18406	0.18406	0.18406	0.18406	0.18406	0.18406	0.18406	0.18406	0.18406	0.18406	0.18406
ABC-ES	-	0.0407021	0.0407021	0.0407021	0.0407021	0.0407021	0.0407021	0.0407021	0.0407021	0.0407021	0.0407021	0.0407021	0.0407021	0.0407021
ABCx+ADVM-m5	-	-	0.18406	0.18406	0.18406	0.18406	0.18406	0.18406	0.18406	0.18406	0.18406	0.18406	0.18406	0.18406
MABC	-	-	-	0.18406	0.18406	0.18406	0.18406	0.18406	0.18406	0.18406	0.18406	0.18406	0.18406	0.18406
ABC+A-DVM	-	-	-	-	0.18406	0.18406	0.18406	0.18406	0.18406	0.18406	0.18406	0.18406	0.18406	0.18406
ABCx-m5	-	-	-	-	-	0.18406	0.18406	0.18406	0.18406	0.18406	0.18406	0.18406	0.18406	0.18406
GBESTABC+A-DVM	-	-	-	-	-	-	0.18406	0.18406	0.18406	0.18406	0.18406	0.18406	0.18406	0.18406
EPSO	-	-	-	-	-	-	-	0.18406	0.18406	0.18406	0.18406	0.18406	0.18406	0.18406
ABC	-	-	-	-	-	-	-	-	0.18406	0.18406	0.18406	0.18406	0.18406	0.18406
ABCx-m1	-	-	-	-	-	-	-	-	-	0.18406	0.18406	0.18406	0.18406	0.18406
GBESTABC	-	-	-	-	-	-	-	-	-	-	0.18406	0.18406	0.18406	0.18406
GBESTABC2+A-DVM	-	-	-	-	-	-	-	-	-	-	-	0.18406	0.18406	0.18406
GBESTABC2	-	-	-	-	-	-	-	-	-	-	-	-	0.18406	0.18406
ABCx+ADVM-m1	-	-	-	-	-	-	-	-	-	-	-	-	-	0.18406
**Zimmerman**	**ABC-ES**	**ABCx+ADVM-m5**	**MABC**	**ABC+A-DVM**	**ABCx-m5**	**GBESTABC+A-DVM**	**EPSO**	**ABC**	**ABCx-m1**	**GBESTABC**	**GBESTABC2+A-DVM**	**GBESTABC2**	**ABCx+ADVM-m1**	**MABC+ADVM**
PSO	4.56246 × 10${}^{-6}$	0.00191853	0.00145742	0.000903768	0.000513593	0.00646495	0.000540484	0.000903629	3.68175 × 10${}^{-5}$	0.00340867	0.00765646	0.00866069	0.000138162	0.00145742
ABC-ES	-	0.000804015	0.000273693	0.000898623	0.0053294	4.97132 × 10${}^{-6}$	0.0314519	0.000898472	0.218961	3.50841 × 10${}^{-5}$	1.51208 × 10${}^{-6}$	9.64117 × 10${}^{-7}$	0.0840264	0.000273693
ABCx+ADVM-m5	-	-	0.267523	0.204729	0.252784	0.0178275	0.0427109	0.211192	0.00320394	0.0260143	0.0955879	0.063022	0.0120503	0.267523
MABC	-	-	-	0.34173	0.104913	0.0273778	0.192421	0.389072	0.106618	0.0348831	0.0217261	0.00258444	0.490923	0.18406
ABC+A-DVM	-	-	-	-	0.152641	0.0291895	0.138743	0.467464	0.167898	0.0351092	0.0833004	0.00778878	0.394688	0.34173
ABCx-m5	-	-	-	-	-	0.00192754	0.367506	0.142265	0.0583064	0.00441018	0.00110716	0.000649672	0.179864	0.104913
GBESTABC+A-DVM	-	-	-	-	-	-	0.0183738	0.0296956	0.000427548	0.400006	0.40357	0.426642	0.00336902	0.0273778
EPSO	-	-	-	-	-	-	-	0.138739	0.0863635	0.0369074	0.186093	0.131925	0.268265	0.192421
ABC	-	-	-	-	-	-	-	-	0.167895	0.0351072	0.0756454	0.00633942	0.394687	0.389072
ABCx-m1	-	-	-	-	-	-	-	-	-	0.00162872	0.00258724	0.00152962	0.220545	0.106618
GBESTABC	-	-	-	-	-	-	-	-	-	-	0.423662	0.449913	0.00883817	0.0348831
GBESTABC2+A-DVM	-	-	-	-	-	-	-	-	-	-	-	0.107119	0.0440778	0.0217261
GBESTABC2	-	-	-	-	-	-	-	-	-	-	-	-	0.0277135	0.00258444
ABCx+ADVM-m1	-	-	-	-	-	-	-	-	-	-	-	-	-	0.490923

**Table 6:** Results of the experiment for all problem instances.

Problem	Algorithm	Mean	Median	Std. Dev	Best	Worst	Problem	Algorithm	Mean	Median	Std. Dev	Best	Worst	Problem	Algorithm	Mean	Median	Std. Dev	Best	Worst
Bukin06	DE	0.97893	0.91922	0.52393	0.33	2.60682	DeVilliersGlasser02	DE	352.642	64.37420	767.121	0.39534	3182.44	SineEnvelope	DE	0.47414	0.47354	0.09202	0.3289	0.69363
	PSO	0.03759	0.04883	0.01611	0.00431	0.05000		PSO	7108.73	9377.49	4258.06	**0.00000**	10647.4		PSO	7.09257	7.26654	0.57119	6.09679	7.86571
	EPSO	**0.00901**	**0.00715**	**0.00690**	0.00057	**0.02419**		EPSO	799.059	6.52004	2538.22	**0.00000**	10467.8		EPSO	4.74872	4.8902	0.89428	3.04664	6.09835
	ABC	0.06535	0.05000	0.03345	0.01909	0.15020		ABC	5.71168	3.20875	6.02096	0.34329	21.70150		ABC	**0.25965**	**0.22372**	0.07091	0.18175	0.39453
	MABC	0.05800	0.05000	0.046600	0.01611	0.21930		MABC	4.12467	2.74194	4.63619	0.24565	15.8204		MABC	3.01514	3.09304	0.30318	2.47955	3.57755
	MABC+ADVM	0.099097	0.071473	0.059937	0.013819	0.261345		MABC+ADVM	3.54775	2.48224	3.45717	0.432356	17.9148		MABC+ADVM	1.99107	1.99038	0.332486	1.22778	2.5769
	GBESTABC	0.18781	0.18014	0.07922	0.05044	0.34700		GBESTABC	8.13107	4.71285	11.61610	0.84988	53.06630		GBESTABC	0.25933	0.23034	0.06796	**0.16192**	0.38841
	GBESTABC2	0.35783	0.33525	0.17333	0.12420	0.63954		GBESTABC2	5.56375	4.42650	4.64582	0.65744	21.59900		GBESTABC2	0.30696	0.30139	0.07077	0.19638	0.43203
	ABC+A-DVM	0.05949	0.04990	0.04087	0.00340	0.15985		ABC+A-DVM	2.53581	1.93048	**2.00250**	0.05803	**7.09280**		ABC+A-DVM	0.30694	0.29637	0.09403	0.19931	0.56087
	GBESTABC+A-DVM	0.17330	0.13761	0.08993	0.04872	0.33291		GBESTABC+A-DVM	6.89254	4.77318	5.30292	1.06621	23.2227		GBESTABC+A-DVM	0.26499	0.25378	**0.05339**	0.18960	**0.37096**
	GBESTABC2+A-DVM	0.32394	0.34000	0.17225	0.09892	0.69858		GBESTABC2+A-DVM	8.36485	7.13058	8.01541	0.40725	32.1687		GBESTABC2+A-DVM	0.29561	0.29025	0.05581	0.19367	0.39693
	ABCXm1	0.027548	0.023434	0.017643	0.00241	0.050081		ABCXm1	3.8484	0.338253	11.4397	5.2 × 10${}^{-5}$	59.3269		ABCXm1	1.06673	1.02163	0.344164	0.475796	1.80824
	ABCXm1+A-DVM	0.026575	0.026393	0.018326	**0.000473**	0.050066		ABCXm1+A-DVM	**2.12372**	**0.338253**	3.37561	4.4 × 10${}^{-5}$	12.0782		ABCXm1+A-DVM	1.1684	1.06626	0.366067	0.514902	1.91655
	ABCXm5	0.029684	0.028866	0.014425	0.002358	0.053062		ABCXm5	3.39312	2.46051	2.57071	0.478018	10.445		ABCXm5	1.03955	1.02833	0.16000	0.691504	1.48731
	ABCXm5+A-DVM	0.029074	0.028134	0.016507	0.00326	0.052951		ABCXm5+A-DVM	3.70145	3.39775	2.66626	0.598359	10.0822		ABCXm5+A-DVM	1.04225	1.06835	0.156475	0.760775	1.33566
Cola	DE	12.44390	12.39020	0.502836	11.77570	13.82660	Griewank	DE	**0.00000**	**0.00000**	**0.00000**	**0.00000**	**0.00000**	Trefethen	DE	−3.29379	**−3.30687**	0.04156	**−3.30687**	−3.14408
	PSO	16.13410	15.30270	2.44014	12.9697	22.06270		PSO	1.34282	1.30004	0.17168	1.10178	1.80049		PSO	−3.08315	−3.17611	0.21692	**−3.30687**	−2.64262
	EPSO	13.42210	13.60100	1.06166	**11.7481**	15.48070		EPSO	0.00910	**0.00000**	0.01379	**0.00000**	0.04426		EPSO	−3.27985	**−3.30687**	0.06253	**−3.30687**	−3.06263
	ABC	**12.05440**	**11.95500**	**0.22993**	11.75370	**12.54730**		ABC	**0.00000**	**0.00000**	**0.00000**	**0.00000**	**0.00000**		ABC	**−3.30687**	**−3.30687**	**0.00000**	**−3.30687**	**−3.30687**
	MABC	12.83970	12.84790	0.443416	12.11390	13.61120		MABC	**0.00000**	**0.00000**	**0.00000**	**0.00000**	**0.00000**		MABC	**−3.30687**	**−3.30687**	**0.00000**	**−3.30687**	**−3.30687**
	MABC+ADVM	12.2932	12.3446	0.327254	11.8035	12.9642		MABC+ADVM	0.002013	0.000020	0.00483	**0.00000**	0.022877		MABC+ADVM	−3.30687	−3.30687	**0.00000**	−3.30687	−3.30687
	GBESTABC	12.22790	12.15260	0.30889	11.79990	13.08850		GBESTABC	**0.00000**	**0.00000**	**0.00000**	**0.00000**	**0.00000**		GBESTABC	**−3.30687**	**−3.30687**	**0.00000**	**−3.30687**	**−3.30687**
	GBESTABC2	12.23520	12.25250	0.28235	11.80430	12.76960		GBESTABC2	**0.00000**	**0.00000**	**0.00000**	**0.00000**	**0.00000**		GBESTABC2	**−3.30687**	**−3.30687**	**0.00000**	**−3.30687**	**−3.30687**
	ABC+A-DVM	12.15250	12.08500	0.232509	11.84320	12.65390		ABC+A-DVM	0.00041	**0.00000**	0.00186	**0.00000**	0.00834		ABC+A-DVM	**−3.30687**	**−3.30687**	**0.00000**	**−3.30687**	**−3.30687**
	GBESTABC+A-DVM	12.28230	12.18300	0.32599	11.81830	13.12510		GBESTABC+A-DVM	**0.00000**	**0.00000**	**0.00000**	**0.00000**	**0.00000**		GBESTABC+A-DVM	**−3.30682**	**−3.30687**	0.00022	**−3.30687**	−3.30588
	GBESTBBC2+A-DVM	12.23630	12.21250	0.29413	11.82890	12.96570		GBESTABC2+A-DVM	**0.00000**	**0.00000**	**0.00000**	**0.00000**	1.43 × 10${}^{-5}$		GBESTABC2+A-DVM	**−3.30687**	**−3.30687**	**0.00000**	**−3.30687**	**−3.30687**
	ABCXm1	12.7074	12.5568	0.759207	11.7718	14.6546		ABCXm1	0.013916	0.013824	0.01168	4.2 × 10${}^{-5}$	0.047391		ABCXm1	−3.24238	−3.20814	0.070179	−3.30687	−3.06263
	ABCXm1+A-DVM	12.8568	13.0618	0.570437	11.8139	14.1411		ABCXm1+A-DVM	0.012559	0.012324	0.010067	1.5 × 10${}^{-5}$	0.037476		ABCXm1+A-DVM	−3.26056	−3.30687	0.079556	−3.30687	−3.06263
	ABCXm5	12.1119	11.9955	0.262208	11.77	12.7076		ABCXm5	0.000775	0.000684	0.000403	0.000213	0.002204		ABCXm5	−3.26755	−3.30687	0.068596	−3.30687	−3.06263
	ABCXm5+A-DVM	12.0595	12.0312	**0.195371**	11.7971	12.5531		ABCXm5+A-DVM	0.000751	0.000633	0.000424	0.000207	0.002318		ABCXm5+A-DVM	−3.297	−3.30687	0.029619	−3.30687	−3.20814
CrossLegTable	DE	−0.26326	−0.08477	0.37832	**−1.00000**	−0.00611	Rastrigin	DE	0.54722	0.49748	0.60175	**0.00000**	1.98992	XinSheYang03	DE	**0.00000**	**0.00000**	**0.00000**	**0.00000**	**0.00000**
	PSO	−0.09723	−0.08283	0.21626	**−1.00000**	−0.00255		PSO	125.84700	126.20500	21.16520	88.1972	160.206		PSO	**0.00000**	**0.00000**	**0.00000**	**0.00000**	**0.00000**
	EPSO	−0.17534	−0.08477	0.28203	**−1.00000**	−0.07959		EPSO	45.76950	51.25510	23.05110	6.96471	99.49550		EPSO	**0.00000**	**0.00000**	**0.00000**	**0.00000**	**0.00000**
	ABC	−0.13062	−0.08493	0.20463	**−1.00000**	−0.08477		ABC	**0.00000**	**0.00000**	**0.00000**	**0.00000**	**0.00000**		ABC	**0.00000**	**0.00000**	**0.00000**	**0.00000**	**0.00000**
	MABC	−0.10630	−0.08477	0.09595	−0.51398	−0.08477		MABC	74.55290	75.18170	8.78129	50.39630	87.9141		MABC	**0.00000**	**0.00000**	**0.00000**	**0.00000**	**0.00000**
	MABC+ADVM	−0.084594	−0.084778	0.000587	−0.084933	−0.082837		MABC+ADVM	6.29867	6.04595	1.80949	2.15974	10.3941		MABC+ADVM	**0.00000**	**0.00000**	**0.00000**	**0.00000**	**0.00000**
	GBESTABC	−0.12994	−0.08477	0.20479	**−1.00000**	−0.07981		GBESTABC	**0.00000**	**0.00000**	**0.00000**	**0.00000**	**0.00000**		GBESTABC	**0.00000**	**0.00000**	**0.00000**	**0.00000**	**0.00000**
	GBESTABC2	−0.08448	−0.08477	**0.00071**	−0.08477	−0.08283		GBESTABC2	**0.00000**	**0.00000**	**0.00000**	**0.00000**	**0.00000**		GBESTABC2	**0.00000**	**0.00000**	**0.00000**	**0.00000**	**0.00000**
	ABC+A-DVM	−0.13061	−0.08493	0.20463	**−1.00000**	−0.08477		ABC+A-DVM	**0.00000**	**0.00000**	**0.00000**	**0.00000**	**0.00000**		ABC+A-DVM	**0.00000**	**0.00000**	**0.00000**	**0.00000**	**0.00000**
	GBESTABC+A-DVM	−0.12355	−0.08477	0.20728	**−1.00000**	−0.00656		GBESTABC+A-DVM	**0.00000**	**0.00000**	**0.00000**	**0.00000**	**0.00000**		GBESTABC2+A-DVM	**0.00000**	**0.00000**	**0.00000**	**0.00000**	**0.00000**
	GBESTABC2+A-DVM	−0.13044	−0.08477	0.20467	**−1.00000**	−0.08283		GBESTABC2+A-DVM	**0.00000**	**0.00000**	**0.00000**	**0.00000**	**0.00000**		GBESTABC+A-DVM	**0.00000**	**0.00000**	**0.00000**	**0.00000**	**0.00000**
	ABCXm1	−0.032665	−0.039932	0.022017	−0.063706	−0.004235		ABCXm1	0.598405	0.171957	0.709102	0.002089	2.10224		ABCXm1	**0.00000**	**0.00000**	**0.00000**	**0.00000**	**0.00000**
	ABCXm1+A-DVM	−0.028716	−0.041177	0.019874	−0.055539	−0.003221		ABCXm1+A-DVM	0.833917	0.824083	0.842761	0.004411	3.21893		ABCXm1+A-DVM	**0.00000**	**0.00000**	**0.00000**	**0.00000**	**0.00000**
	ABCXm5	−0.049196	**−0.006641**	0.178485	**−1.00000**	−0.001788		ABCXm5	0.029805	0.028714	0.009302	0.014585	0.05643		ABCXm5	**0.00000**	**0.00000**	**0.00000**	**0.00000**	**0.00000**
	ABCXm5+A-DVM	**−0.020866**	−0.007002	0.031149	−0.084778	−0.000575		ABCXm5+A-DVM	0.034629	0.029712	0.017951	0.01176	0.091563		ABCXm5+A-DVM	**0.00000**	**0.00000**	**0.00000**	**0.00000**	**0.00000**

**Table 7:** Results of the experiment for all problem instances.

Problem	Algorithm	Mean	Median	Std. Dev	Best	Worst	Problem	Algorithm	Mean	Median	Std. Dev	Best	Worst	Problem	Algorithm	Mean	Median	Std. Dev	Best	Worst
CrownedCross	DE	0.00173	0.00117	0.00347	**0.00010**	0.01635	Rosenbrock	DE	32.86880	25.58250	24.11440	7.49392	86.07950	Whitley	DE	0.01388	1.20 × 10${}^{-5}$	0.01925	**0.00000**	0.03945
	PSO	0.01609	0.00120	0.01884	**0.00010**	0.03909		PSO	163741	122694	107137	31992.8	449777		PSO	0.03286	0.03945	0.04100	**0.00000**	0.15783
	EPSO	0.00108	0.00117	0.00033	**0.00010**	0.00125		EPSO	8.20397	8.59076	2.84563	3.16795	13.15980		EPSO	0.00591	**0.00000**	0.01445	**0.00000**	0.03945
	ABC	0.00117	**0.00010**	**0.00000**	0.00117	**0.00117**		ABC	**0.91220**	**0.15323**	**1.44928**	**0.01712**	**4.69119**		ABC	**0.00003**	**0.00000**	0.00011	**0.00000**	0.00050
	MABC	0.00113	**0.00010**	0.0002	0.00027	**0.00117**		MABC	44.68880	27.22880	29.94650	23.93810	112.734		MABC	0.00203	**0.00000**	0.00881	**0.00000**	0.03945
	MABC+ADVM	0.001186	0.00118	3.1 × 10${}^{-5}$	0.001177	0.001349		MABC+ADVM	13.5615	5.57401	21.179	0.055279	78.1312		MABC+ADVM	38.1889	20.0484	44.4305	1.03001	182.301
	GBESTABC	0.00108	**0.00010**	0.00033	**0.00010**	0.00120		GBESTABC	1.70578	1.09851	1.85263	0.08547	6.11791		GBESTABC	0.00757	1.37 × 10${}^{-5}$	0.01276	**0.00000**	0.03945
	GBESTABC2	0.00118	**0.00010**	1.13 × 10${}^{-5}$	0.00117	0.00120		GBESTABC2	3.18224	2.5852	2.93224	0.35930	13.16040		GBESTABC2	0.00236	0.00015	0.00852	**0.00000**	0.03845
	ABC+A-DVM	**0.00105**	**0.00010**	0.00032	**0.00010**	**0.00117**		ABC+A-DVM	2.55989	1.04791	4.37552	0.02863	19.18300		ABC+A-DVM	0.00064	**0.00000**	0.00226	**0.00000**	0.01009
	GBESTABC+A-DVM	0.00113	**0.00010**	0.00024	**0.00010**	0.00125		GBESTABC+A-DVM	3.46204	1.48614	4.40623	0.04360	13.87970		GBESTABC+A-DVM	0.00618	**0.00000**	0.01435	**0.00000**	0.03945
	GBESTABC2+A-DVM	0.00118	**0.00010**	**0.00000**	0.00117	0.00120		GBESTABC2+A-DVM	4.55452	3.8697	3.73467	0.11879	14.8367		GBESTABC2+A-DVM	0.00062	0.00022	**0.00095**	**0.00000**	**0.00379**
	ABCXm1	0.011439	0.002754	0.010681	0.001389	0.028141		ABCXm1	72.0576	82.1424	28.3858	7.44934	109.364		ABCXm1	85.0402	57.9288	67.5181	26.3005	270.85
	ABCXm1+A-DVM	0.008005	0.002100	0.00942	0.001622	0.030850		ABCXm1+A-DVM	64.3322	77.9291	32.1162	2.19893	106.097		ABCXm1+A-DVM	62.608	44.1697	51.6055	15.8966	269.044
	ABCXm5	0.018339	0.015057	0.01484	0.0001	0.055938		ABCXm5	22.21	19.2556	9.54515	9.69884	47.4673		ABCXm5	59.9045	58.6329	14.0889	38.66390	102.5700
	ABCXm5+A-DVM	0.025878	0.014298	0.032853	0.00118	0.173876		ABCXm5+A-DVM	18.6672	14.0424	15.1627	5.32653	82.7050		ABCXm5+A-DVM	61.429	59.2038	15.6881	37.0757	100.947
Damavandi	DE	2.00000	2.00000	**0.00000**	2.00000	2.00000	Schwefel06	DE	**0.00000**	**0.00000**	**0.00000**	**0.00000**	**0.00000**	Zimmerman	DE	0.38522	0.69869	0.35633	**0.00000**	0.70133
	PSO	2.00000	2.00000	**0.00000**	2.00000	2.00000		PSO	**0.00000**	**0.00000**	**0.00000**	**0.00000**	**0.00000**		PSO	715.1750	1300	663.34500	**0.00000**	1300.000
	EPSO	1.90000	2.00000	0.44721	**0.00000**	2.00000		EPSO	**0.00000**	**0.00000**	**0.00000**	**0.00000**	**0.00000**		EPSO	0.17464	**0.00000**	0.31035	**0.00000**	0.69858
	ABC	2.00000	2.00000	**0.00000**	2.00000	2.00000		ABC	0.17159	0.10386	0.17989	0.00056	0.59950		ABC	0.00037	**0.00000**	0.00126	**0.00000**	0.00569
	MABC	2.00000	2.00000	**0.00000**	2.00000	2.00000		MABC	**0.00000**	**0.00000**	**0.00000**	**0.00000**	**0.00000**		MABC	**0.00000**	**0.00000**	**0.00000**	**0.00000**	**0.00000**
	MABC+ADVM	1.61113	2.00000	0.778219	0.001555	2.00000		MABC+ADVM	0.123341	0.076095	0.180687	0.002949	0.767581		MABC+ADVM	0.000315	1.7 × 10${}^{-5}$	0.001023	0.000000	0.005673
	GBESTABC	1.76059	2.00000	0.60220	0.00611	2.00000		GBESTABC	0.13196	0.11892	0.08783	0.01193	0.28331		GBESTABC	0.10626	0.00053	0.25554	**0.00000**	0.69923
	GBESTABC2	1.82772	2.00000	0.53654	0.02502	2.00000		GBESTABC2	0.13886	0.12197	0.10182	0.01223	0.42978		GBESTABC2	0.07062	0.00050	0.21487	5.96 × 10${}^{-5}$	0.69904
	ABC+A-DVM	1.80056	2.00000	0.61387	0.00258	2.00000		ABC+A-DVM	0.11728	0.04898	0.17793	0.00272	0.72679		ABC+A-DVM	0.00041	3.27 × 10${}^{-5}$	0.00097	**0.00000**	0.00325
	GBESTABC+A-DVM	1.81787	2.00000	0.56099	0.11335	2.00000		GBESTABC+A-DVM	0.09749	0.10197	0.04416	0.02492	0.16431		GBESTABC+A-DVM	0.10543	0.00035	0.25582	**0.00000**	0.69921
	GBESTABC2+A-DVM	**1.74073**	2.00000	0.63782	0.00028	2.00000		GBESTABC2+A-DVM	0.14695	0.09799	0.12129	0.02657	0.46299		GBESTABC2+A-DVM	0.03531	0.00020	0.15612	1.40 × 10${}^{-5}$	0.69862
	ABCXm5	2.00000	2.00000	**0.00000**	2.00000	2.00000		ABCXm5	**0.00000**	**0.00000**	**0.00000**	**0.00000**	**0.00000**		ABCXm5	0.232862	**0.00000**	0.329315	**0.00000**	0.698586
	ABCXm5+A-DVM	2.00000	2.00000	**0.00000**	2.00000	2.00000		ABCXm5+A-DVM	**0.00000**	**0.00000**	**0.00000**	**0.00000**	**0.00000**		ABCXm5+A-DVM	0.349292	0.349292	0.349292	**0.00000**	0.698586
	ABCXm1	2.00000	2.00000	**0.00000**	2.00000	2.00000		ABCXm1	**0.00000**	**0.00000**	**0.00000**	**0.00000**	**0.00000**		ABCXm1	0.256146	**0.00000**	0.336642	**0.00000**	0.698581
	ABCXm1+A-DVM	1.93333	2.00000	0.35901	**0.00000**	2.00000		ABCXm1+A-DVM	**0.00000**	**0.00000**	**0.00000**	**0.00000**	**0.00000**		ABCXm1+A-DVM	0.326004	**0.00000**	0.348513	**0.00000**	0.698581

## Abbreviations

The following abbreviations are used in this manuscript:

SI	Swarm Intelligence
ABC	Artificial Bee Colony
ABC-ES	Artificial Bee Colony with Evolution Strategies
A-DVM	Adaptive Decision Variable Matrix
PSO	Particle Swarm Optimization
DE	Differential Evolution
SPD	Spatial Population Diversity
HPD	Healthy Population Diversity
MABC	Modified Artificial Bee Colony
GBESTABC	Global Best Artificial Bee Colony
